# Heterologous versus homologous COVID-19 booster vaccinations for adults: systematic review with meta-analysis and trial sequential analysis of randomised clinical trials

**DOI:** 10.1186/s12916-024-03471-3

**Published:** 2024-06-24

**Authors:** Mark Aninakwah Asante, Martin Ekholm Michelsen, Mithuna Mille Balakumar, Buddheera Kumburegama, Amin Sharifan, Allan Randrup Thomsen, Steven Kwasi Korang, Christian Gluud, Sonia Menon

**Affiliations:** 1 Copenhagen Trial Unit, Centre for Clinical Intervention Research, The Capital Region, Copenhagen University Hospital – Rigshospitalet, Copenhagen, Denmark; 2 Department of Pharmaceutical Care, Sina Hospital, Tehran University of Medical Sciences, Tehran, Iran; 3 Department of Immunology and Microbiology, Faculty of Health and Medical Sciences, University of Copenhagen, Copenhagen, Denmark; 4 Department of Pediatrics, Children’s Hospital Los Angeles, Los Angeles, CA USA; 5 Department of Regional Health Research, The Faculty of Health Sciences, University of Southern Denmark, Odense, Denmark; 6 Epitech Research, Brussels, Belgium

**Keywords:** COVID-19 vaccines, Booster immunisation, Heterologous immunity, Homologous immunity, Vaccine efficacy, Vaccine safety

## Abstract

**Background:**

To combat coronavirus disease 2019 (COVID-19), booster vaccination strategies are important. However, the optimal administration of booster vaccine platforms remains unclear. Herein, we aimed to assess the benefits and harms of three or four heterologous versus homologous booster regimens.

**Methods:**

From November 3 2022 to December 21, 2023, we searched five databases for randomised clinical trials (RCT). Reviewers screened, extracted data, and assessed bias risks independently with the Cochrane risk-of-bias 2 tool. We conducted meta-analyses and trial sequential analyses (TSA) on our primary (all-cause mortality; laboratory confirmed symptomatic and severe COVID-19; serious adverse events [SAE]) and secondary outcomes (quality of life [QoL]; adverse events [AE] considered non-serious). We assessed the evidence with the GRADE approach. Subgroup analyses were stratified for trials before and after 2023, three or four boosters, immunocompromised status, follow-up, risk of bias, heterologous booster vaccine platforms, and valency of booster.

**Results:**

We included 29 RCTs with 43 comparisons (12,538 participants). Heterologous booster regimens may not reduce the relative risk (RR) of all-cause mortality (11 trials; RR 0.86; 95% CI 0.33 to 2.26; *I*^2^ 0%; very low certainty evidence); laboratory-confirmed symptomatic COVID-19 (14 trials; RR 0.95; 95% CI 0.72 to 1.25; *I*^2^ 0%; very low certainty); or severe COVID-19 (10 trials; RR 0.51; 95% CI 0.20 to 1.33; *I*^2^ 0%; very low certainty). For safety outcomes, heterologous booster regimens may have no effect on SAE (27 trials; RR 1.15; 95% CI 0.68 to 1.95; *I*^2^ 0%; very low certainty) but may raise AE considered non-serious (20 trials; RR 1.19; 95% CI 1.08 to 1.32; *I*^2^ 64.4%; very low certainty). No data on QoL was available. Our TSAs showed that the cumulative *Z* curves did not reach futility for any outcome.

**Conclusions:**

With our current sample sizes, we were not able to infer differences of effects for any outcomes, but heterologous booster regimens seem to cause more non-serious AE. Furthermore, more robust data are instrumental to update this review.

**Supplementary Information:**

The online version contains supplementary material available at 10.1186/s12916-024-03471-3.

## Background

Severe respiratory syndrome coronavirus 2 (SARS-CoV-2) is the pathogen that causes coronavirus disease (COVID-19). Despite the official end of the public health emergency declaration on 5 May 2023, SARS-CoV-2 continues to infect people across the world, with vaccination remaining one of the most important protective measures against COVID-19 [1, 2].

Between 31 July and 27 August 2023, more than 1.4 million new COVID-19 patients and over 1800 deaths were reported globally underscoring the need for ongoing close monitoring of circulating SARS-CoV-2 variants closely [1]. Presently, a number of variants are tracked by WHO, including two variants of interest (VOIs) (XBB.1.5 and XBB.1.16) and a number of variants under monitoring (VUMs) [1]. Significant progress in the handling of the COVID-19 epidemic has already been made as nearly every country has implemented vaccination policies, which has resulted in major reductions in the occurrence of severe disease, hospitalisations, and mortality [2].

Despite fewer severely diseased and fewer deaths worldwide today, there are concerns about reduced protection because of waning immunity and the appearance of newly emerging variants [3]. Currently, the Strategic Advisory Group of Experts on Immunisation recommends healthy adults over the age of 18 years are to receive one booster dose after primary vaccine series, whilst individuals with the greater risk of severe disease and death (older adults, pregnant persons, and people with immunocompromised conditions) are recommended an additional booster dose [4].

Using heterologous vaccine platforms can be an alternative strategy to homologous vaccine platforms to maximise booster vaccine impact in the event of limited supplies. It is unclear whether a heterologous boosting regimen may provide higher vaccine effectiveness than homologous booster vaccines. Two meta-analyses including randomised clinical trials and observational studies suggest that heterologous booster doses have a higher protection against symptomatic COVID-19 and severe COVID-19 compared with or to homologous booster doses [5, 6] whilst a ‘living meta-analysis’ also including randomised clinical trials and observational studies does not [7].

The objective of this systematic review is to compare the vaccine benefits and harms between three or four dose heterologous boosters using different vaccine platforms or intra-platform variations versus homologous booster regimens in randomised trials only to help inform public health policies.

## Methods

Recognising the needs of COVID-19 vaccine research and the identification of trials on heterologous versus homologous booster regimens as an area of public health interest necessitating evidence synthesis, we performed this specific review of pairwise comparison of heterologous versus homologous boosters in randomised clinical trials. This was performed within the framework of our living systematic review, the methodology of which is thoroughly discussed elsewhere [8], and the protocol registered in PROSPERO (CRD42020178787). This systematic review was reported in accordance with the Preferred Reporting Items for Systematic Reviews and Meta-Analysis [9] (Additional file: PRISMA checklist**)** and the implementation of this review followed the recommended procedures as specified in the Cochrane Handbook of Systematic Reviews of Interventions [10].

### Search strategy and trial inclusion criteria

This updated review follows a two-step approach. As for the first living systematic review, the literature searches were conducted on a biweekly basis, from 3 November 2022 to 21 December 2023 using Medline, Cochrane Central Register of Controlled Trials, Embase, Latin American and Caribbean Health Sciences Literature, and Science Citation Index Expanded to identify newly published trials following the initial search strategy and eligibility criteria (for more ample information on the search strategy and study inclusion, please refer to the protocol (Additional file: Additional search Strategy]). After identifying eligible randomised clinical trials for our original research on the efficacy of all COVID-19 vaccines in relation to all-cause mortality, safety, and vaccine efficacy, we employed a specific search strategy tailored to our present research question (Additional file: Additional search strategy). As a quality control measure, we also conducted a snowball search to identify any potential missed trials [11]. All randomised clinical trials reporting on a third or fourth heterologous booster vaccine versus either a third or fourth homologous booster vaccine were included. In instances where it was not possible to determine whether the intervention arm used a heterologous or homologous booster vaccine, and no clarification was provided by the authors, the trial was excluded. Also, only full booster doses between both arms were compared, in instances when boosters between both arms only compared half doses to full doses, the trial was excluded. Trials with mixed primary series in the heterologous arm were excluded. Furthermore, trials reporting exclusively on immunogenicity, along with trials comparing different types of heterologous booster vaccines or heterologous third booster to a placebo were also excluded. Trials that included open-label cohorts with no randomisation of the participants were excluded.

### Data analysis

#### Outcomes

The vaccine efficacy outcomes included the primary outcomes, all-cause mortality, prevention of laboratory-confirmed symptomatic COVID-19, severe symptoms associated with COVID-19, and serious adverse events (SAE) [8]. Whenever participants were noted to have (laboratory-confirmed) COVID-19 symptoms, we classified it as symptomatic COVID-19. Conversely, if participants were hospitalised due to severe COVID-19 symptoms, we defined it as severe COVID-19. Secondary outcomes were health-related quality of life and adverse events (AE) considered not serious [8]. We used the trial results reported at maximum follow-up for each specific abovementioned outcome and used intention-to-treat data if provided by the trialist.

#### Data extraction and risk of bias assessment

Two independent authors conducted the screening, data extraction, quality assessment, and GRADE assessment for each eligible trial following the Cochrane risk of bias tool—version 2 and the procedure described in our protocol. If three domains were assigned a ‘some concern’ assessment, then the trial was graded at ‘high risk of bias’. Any discrepancies were resolved by consensus and authors were contacted to clarify uncertainties and provide additional context, including available data stratified by older adults.

#### Statistical synthesis

We performed meta-analysis using STATA 17 for Windows (StataCorp, College Station, TX, USA, 2021) and analysed data with the meta command for meta-analysis. For the trial sequential analysis (TSA), we used version 0.9.5.10 beta (TSA 2017) [12]. To quantify the strength of associations between booster vaccines and vaccine efficacy and safety outcomes, we employed relative risk (RR). The risk ratio was computed by dividing the risk observed in the heterologous vaccine regimen group by the risk in the homologous vaccine regimen group, and the 95% confidence intervals (CI) for the risk ratio was used to determine the precision of the estimated associations. With a view to avoiding attributing excessive weight to the control groups in the meta-analysis, we divided both the numerator and the denominator of the control group by the number of intervention groups whenever the same control group was used in a trial to compare different intervention groups. To account for potential heterogeneity amongst the trials, random-effects DerSimonian and Laird models were applied [13, 14]. In addition, the fixed-effect meta-analysis (Mantel–Haenszel method) was assessed separately and the most conservative point estimate of the two reported [15, 16]. We also post hoc applied Peto’s odds ratio (OR) due to very few outcomes in some comparisons.

Assessment of heterogeneity within and between study groups was conducted using the Cochrane Q test, with a significance level of *p* < 0.1 indicating the presence of heterogeneity [10]. The *I*^2^ statistic, as described by Higgins and Thompson was employed to estimate the percentage of observed between-study variability due to heterogeneity, as opposed to chance [17]. This statistic ranges from 0 to 100%, with values of 0 to 40% representing moderate heterogeneity, 30 to 60% moderate heterogeneity, 50 to 90% substantial heterogeneity, and 75 to 100% considerable heterogeneity [10].

Furthermore, we performed a subgroup analysis based on the risk of biases to examine the effect of potential biases on the risk ratio. The variable was categorised as low risk of bias compared to some concerns/high risk of bias, allowing us to discern any differential effects on the overall results. Moreover, we conducted subgroup analyses based on the follow-up time: studies with follow-up periods of 3 months and under were compared to those with follow-up periods of above 3 months. Additionally, we compared vaccine regimens with three doses against those with four doses to explore differences in their risk ratios. As different vaccine booster platforms use distinct mechanisms to elicit immune responses [18], which may lead to varying efficacy and safety profiles [19], we also conducted a subgroup analysis to compare differences in risk ratios between boosters with different vaccine platforms, including inactivated, protein-based, viral vectored, and mRNA-based boosters. Furthermore, we investigated the variation in risk ratios for vaccine efficacy outcomes between trials from 2023 and those from 2022, thereby allowing us to consider the potential influence of the predominance of XBB subvariants towards the end of 2022 and 2023. Also, we conducted a subgroup analysis by immunocompromised status as immunocompromised individuals may not have a robust immune response to COVID-19 vaccines compared to those without an immunocompromised condition [20]. Initially, our plan was to conduct a subgroup analysis by categorising adults into younger and older age groups; however, we were constrained by the absence of disaggregated data. Additionally, as an increase in inoculation interval times may impact vaccine efficacy and possibly safety outcomes [21], we aimed to investigate the impact of different inoculation interval times on vaccine efficacy and safety outcomes using a 12-week cutoff [22]. Nevertheless, inconsistent reporting and a lack of interpretable data due to large ranges of inoculation intervals prevented us from conducting these planned subgroup analyses. To capture more recent trials comparing vaccine valency, monovalent vaccine boosters to multivalent vaccine boosters (bivalent and tetravalent vaccine boosters) using heterologous and homologous vaccine boosters, we have also conducted a subgroup analysis. By conducting these subgroup analyses, we aimed to assess the differential effect on risk ratios and their associated heterogeneity.

We conducted the TSAs to control risks of type I and type II errors [23–25]. To assess publication bias, a visual inspection of the funnel plots was conducted and the Egger statistical test performed when an outcome had at least 10 trials [10].

#### Summary of findings and assessment of certainty

We used the Grading of Recommendations, Assessment, Development, and Evaluation (GRADE) profiler Guideline Development Tool to create the summary of findings tables (GRADEpro GDT https://www.gradepro.org/). We created a summary of findings tables including each of the prespecified outcomes (all-cause mortality, vaccine efficacy, serious adverse events, health-related quality of life, and non-serious adverse events) (Table 1: GRADE assessment). We used the five GRADE considerations (bias risk of the trials, consistency of effect, imprecision, indirectness, and publication bias). We assessed imprecision using trial sequential analysis [8, 26, 27].

**Table 1:**
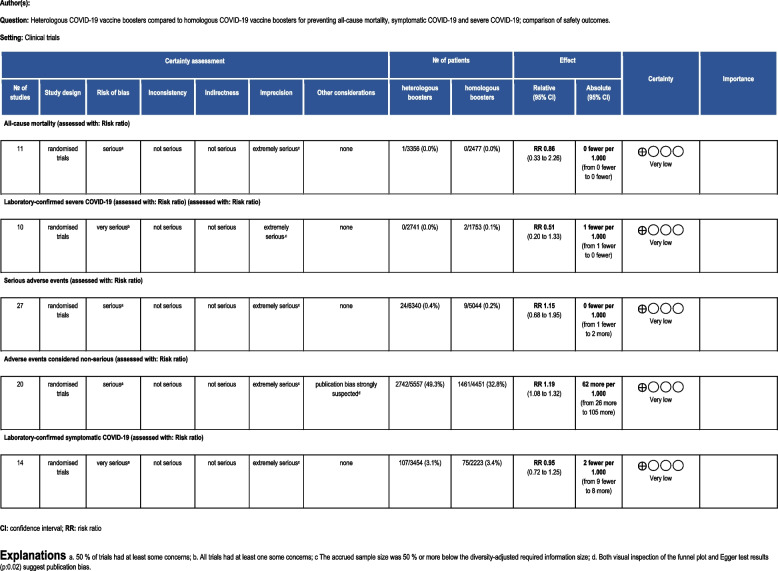
GRADE assessment

## Results

### Trial characteristics

Out of 29,145 abstracts screened by the initial search, 28,044 were excluded after abstract screening. Following a full-text review of 1,101 studies, 601 were excluded based on our inclusion and exclusion criteria. Ultimately, 500 trials met our criteria for the initial research question, of which 29 trials conducted in Europe, North America, Asia, and Latin America were retained in the final analysis of this specific research question. See the PRISMA flow diagram for more details about reasons for exclusion (Additional file: PRISMA flow chart).

In total, 12,538 participants provided data for our predefined meta-analyses. All participants were adults (≥ 18 years) and all trials included older adults (either ≥ 60 or ≥ 65 years) except for four trials [28–31] while five trials exclusively included immunocompromised participants [32–36]. None of the trials included pregnant women. One trial exclusively included healthy older adults (≥ 60 years) [37]. Most trials assessed a third dose heterologous booster vaccine compared with a third dose homologous booster vaccine [28–53] while four trials compared a fourth heterologous booster with a fourth homologous booster [47, 54–56]. The included heterologous booster vaccines encompassed viral-vectored, mRNA, protein subunit, or inactivated virus platforms (Table 2: Trials’ characteristics). Follow-up of participants varied from 7 to 365 days after randomisation for all outcomes. Inoculation intervals between the 2nd and 3rd dose, when reported, ranged from 8 to 43 weeks and 28 to 37 weeks between the 3rd dose and 4th dose (Table 2: Trials’ characteristics).

**Table 2:** Trials’ characteristics

First author surname	Number of doses	Clinical registry	Country of participants	Age and health status of eligible participants	Inoculation interval between second/third dose and heterologous booster dose	Heterologous booster (prime series + booster)	Heterologous booster vaccine platform	Homologous vaccine regimen (3× or 4×)	Arms (number of participants randomised)	Arms (% male)	Heterologous arm: age in years (median and IQR) and (mean and SD)	Homologous arm: age in years (median and IQR) and (mean and SD)
Ahi 2022 [48]	3	IRCT20210206050259N4	Iran	Aged 18 years or older and healthy	10 to 28 weeks	(2× BBIBP-CorV) + FAKHRAVAC	Inactivated	BBIBP-CorV	Heterologous (*N* = 216); homologous (*N* = 219)	Heterologous (84.3%); homologous (85.8%)	41 (IQR: 16.5)	42 (IQR: 18)
Akahata 2023 [51]	2	jRCT2051210164	Japan	Aged 18 years or older and healthy	24 to 48 weeks	(2× BNT162b2) + VLPCOV-01	mRNA	BNT162b2	Heterologous (*N* = 20); Homologous (*N* = 20)	Heterologous (50%); homologous (65%)	54.1 (SD: 5.63)	59.7 (SD: 2.5)
Aliabadi 2023 [36]	2	IRCT20140818018842N24	Iran	Auto-HSCT recipients more than 18 years old	4 weeks	(2× RBD-TT-conjugated COVID-19 vaccine) + inactivated vaccine	Inactivated	RBD-TT-conjugated COVID-19 vaccine	Heterologous (*N* = 31); homologous (*N* = 30)	Heterologous (74%); homologous (60%)	50 (SD: 11)	51 (SD: 11)
Bonelli 2022 [32]	3	EudraCT: 2021-002348-57	Austria	Aged 18 years or older, with chronic inflammatory rheumatic/neurologic diseases under rituximab therapy	8 weeks	(2× BNT162b2 or mRNA-1273) + ChAdOx1 nCoV-19 *	Viral vector	BNT162b2 or mRNA-1273	Heterologous (*N* = 30); homologous (*N* = 30)	Heterologous: (33.3%); homologous: (17.9%)	60.9 (SD: 15.0)	58.9 (SD: 18.4)
Clemens 2022a [46]	3	RBR–9nn3scw	Brazil	Aged 18 years or older	26 weeks	(2× Coronavac) + Ad26.COV2-S	Viral vector	CoronaVac	Heterologous (*N* = 306); homologous (*N* = 290)	Heterologous (38.6%); homologous (41.3%)	59 (IQR: 22–98)	58 (IQR: 21–95)
Clemens 2022b [46]	3	RBR–9nn3scw	Brazil	Aged 18 years or older	26 weeks	(2× Coronavac) + BNT162b2	mRNA	CoronaVac	Heterologous (*N* = 340); homologous (*N* = 290)	Heterologous (38.7%); homologous (41.3%)	61 (IQR: 21–95)	58 (IQR: 21–95)
Clemens 2022c [46]	3	RBR–9nn3scw.	Brazil	Aged 18 years or older	26 weeks	(2× Coronavac) + ChAdOx1 nCoV-19	Viral vector	CoronaVac	Heterologous (*N* = 304); homologous (*N* = 290)	Heterologous (39.5%); homologous (41.3%)	60 (IQR: 21–96)	58 (IQR: 21–95)
Corominas 2023 [38]	3	NCT05142553	Spain	Aged 18 years or older	43 weeks	(2× BNT162b2) + PHH-1V	Protein subunit	BNT162b2	Heterologous (*N* = 522); homologous (*N* = 260)	Heterologous (36.6%); homologous (36.9%)	42.0 (IQR: 19.0–76.0)	40.0 (IQR: 20.0–74.0)
Fadlyana 2023a [44]	3	INA-GO0HLGB	Indonesia	Aged 18 years or older and healthy	13 to 39 weeks	(2× CoronaVac) + ChAdOx1-S	Viral vector	CoronaVac	Heterologous (*N* = 193); homologous (*N* = 192)	Heterologous (57 %); homologous (56%)	43.4 (SD: 15.0)	42.8 (SD: 15.0)
Fadlyana 2023b [44]	3	INA-GO0HLGB	Indonesia	Aged 18 years or older and healthy	13 to 39 weeks	(2× CoronaVac) + BNT162b2	mRNA	CoronaVac	Heterologous (*N* = 193); homologous (*N* = 192)	Heterologous (50 %); homologous (56%)	45·0 (SD:14.3)	42.8 (SD: 15.0)
Hannawi 2023 [52]	3	NCT05323461	Dubai	Aged 18 years or older and healthy	12 to 96 weeks	(2× BNT162b2)+SCTV01E	Protein subunit	BNT162b2	Heterologous (*N* = 148); homologous (*N* = 149)	Heterologous (100%); homologous (100%)	26.0 (IQR:18–45); 27.4 (SD:6.05)	29.0 (IQR: 18–49); 29.4 (SD:7.32)
Hannawi 2023 [53]	3	NCT05323461	Dubai	Aged 18 years or older and healthy	12 to 96 weeks	(2× BBIBP-CorV)+SCTV01E	Protein subunit	BBIBP-CorV	Heterologous (*N* = 446); homologous (*N* = 451)	Heterologous (99.1%); homologous (100%)	29 (IQR: 18–57); 30.1 (SD: 7.39)	29.5 (IQR: 20–50); 30.4 (SD: 7.57)
Jin 2023 [37]	3	NCT04952727	China	Aged 60 years or older and healthy	20 weeks	(2× CoronaVac) + Ad5-nCOV	Viral vector	CoronaVac	Heterologous (*N* = 99); homologous (*N* = 100)	Heterologous (65.7 %); homologous (60%)	66.0 (IQR: 64.0–70.0); 66.6 (SD: 4.6)	66.0 (IQR: 64.0, 69.0); 66.8 (SD: 3.8)
Kaabi 2022 [40]	3	NCT05069129	UAE	Aged 18 years or older and healthy	17 to 39 weeks	(2× BBIBP-CorV) + NVSI-06-08	Protein subunit	BBIBP-CorV	Heterologous (*N* = 896); homologous (*N* = 904)	Heterologous (96.7 %); homologous (95.2%)	7–9 months: 33.8 (IQR: 19.2–66.8); 34.8 (SD:8.4)	7–9 months: 34.1 (IQR: 18.7–60.5); 34.6 (SD:8.3)
Kaabi 2023 [56]	4	NCT05293548	UAE	Aged 18 years or older and healthy	37 weeks	(3× BBIBP-CorV) + NVSI-06-09	Protein subunit	BBIBP-CorV	Heterologous (*N* = 260); homologous (*N* = 256)	Heterologous (93.9%); homologous (95.3%)	36 (IQR: 19–62); 37.2 (SD: 9.1)	36.5 (IQR: 19–63); 37.3 (SD:8.7)
Kulkarni 2023a [50]	2	CTRI/2022/04/042017	India	Aged 18 years or older and healthy	37 weeks	(2× ChAdOx1 nCoV-19) + SII-NVX-CoV2373	Protein subunit	ChAdOx1 nCoV-1	Heterologous (*N* = 92); homologous (*N* = 94)	Heterologous (44.6%); homologous (45.7%)	36.0 (SD: 11.04)	35.7 (SD: 9.56)
Kulkarni 2023ab [50]	2	CTRI/2022/04/042017	India	Aged 18 years or older and healthy	42 weeks	(2× BBV152) + SII-NVX-CoV2373	Protein subunit	BBV152	Heterologous (*N* = 92); homologous (*N* = 94)	Heterologous (69.6%); homologous (59.6%)	38.0 (SD: 14.13)	35.8 (SD: 14.44)
Launay 2022a [43]	3	NCT05124171	France	Aged 18 years and older and healthy or with a medically stable condition	25 weeks	(BNT162b2 2×) + SanofiGSK D614	Protein subunit	BNT162b2	Heterologous (*N* = 85); homologous (*N* = 41)	Heterologous (61.8%); homologous (52.6%)	40.2 (SD: 13.5)	40.4 (SD: 13.9)
Launay 2022b [43]	3	NCT05124171	France	Aged 18 years and older and healthy or with a medically stable condition	24 weeks	(BNT162b2 2×) + SanofiGSK B.1.351	Protein subunit	BNT162b2	Heterologous (*N* = 80); homologous (*N *= 41)	Heterologous (64.8%); homologous (52.6%)	41.4 (SD: 11.3)	40.4 (SD:13.9)
Leung 2023a [45]	3	NCT05057169	Hong Kong	Aged 18 years or older and healthy	33 weeks	(2× CoronaVac) + BNT162b2	mRNA	CoronaVac	Heterologous (*N* = 186); homologous (*N* = 178)	Heterologous (46%); homologous (53%)	52 (IQR 43–58); 51 (SD: 11)	58 (IQR: 50–64); 55 (SD: 12)
Leung 2023b [45]	3	NCT05057169	Hong Kong	Aged 18 years or older and healthy	32 weeks	(2× BNT162b2) + CoronaVac	Inactivated	BNT162b2	Heterologous (*N* = 202); homologous (*N* = 204)	Heterologous (57%); homologous (48%)	56 (IQR 47–60); 53 (SD: 11)	53 (IQR: 46–59); 52 (SD: 12)
Li 2022 [29]	3	NCT04892459	China	Aged 18–59 years and healthy	14 weeks	(2× CoronaVac) + Convidecia	Viral vector	CoronaVac	Heterologous (*N* = 100); homologous (*N* = 100)	Heterologous (60.4%); homologous (62.8%)	47.0 (IQR: 40.3–51.0)	47.0 (IQR:41.0, 52.0)
Mrak 2022 [33]	3	EudraCT No: 2021-002693-10.	Austria	Aged 18 years or older and under immunosuppressive therapy	14 weeks	(2× BNT162b2) + ChAdOx1 nCoV-19	Viral vector	mRNA (BNT162b2 or mRNA-1273)	Heterologous (*N* = 25); homologous (*N* = 26)	Heterologous (68.2%); homologous (58.3%)	61.2 (SD: 14.9)	63.4 (SD: 1.4)
Munro 2021a [39]	3	ISRCTN73765130	UK	Aged 30 years or older and healthy	11 weeks	(2× ChAd) + NVX-CoV2373	Protein subunit	ChAd0x-nCov19	Heterologous (*N* = 115); homologous (*N* = 111)	Heterologous (47%); homologous (51.4%)	65.3 (IQR: 52.6–74.1); 63.5 (SD: 13.7)	67.8 (IQR: 52.2–75.7); 63.7 (SD: 14.1)
Munro 2021b [39]	3	ISRCTN73765130	UK	Aged 30 years or older and healthy	14 weeks	(2× BNT162b2) + VLA2001	Inactivated	BNT162b2	Heterologous (*N* = 110); homologous (*N* = 110)	Heterologous (46.4%); homologous (44.5%)	61.2 (IQR: 46.2–77.7); 60.9 (SD: 18.1)	64.2 (IQR: 49.8–77.4); 62.6 (SD: 16.9)
Munro 2021c [39]	3	ISRCTN73765130	UK	Aged 30 years or older and healthy	15 weeks	(2× BNT162b2) + Ad26.COV2.S	Viral vector	BNT162b2	Heterologous (*N* = 106); homologous (*N* = 110)	Heterologous (43,4%); homologous (44.5%)	61.6 (IQR: 49.2–78.3); 62 (SD: 17.4)	64.2 (IQR: 49.8–77.4); 62.6 (SD: 16.9)
Munro 2021d [39]	3	ISRCTN73765130	UK	Aged 30 years or older and healthy	14 weeks	(2× BNT162b2) + mRNA1273	mRNA	BNT162b2	Heterologous (*N* = 111); homologous (*N* = 110)	Heterologous (43.2%); homologous (52.7 %)	65.0 (IQR: 50.3–75.5); 63.0 (SD: 15.3)	64.4 (IQR: 47.7–78.2); 62.6 (SD: 17.3)
Munro 2021e [39]	3	ISRCTN73765130	UK	Aged 30 years or older and healthy	14 weeks	(2× BNT162b2) + CVnCOV	mRNA	BNT162b2	Heterologous (*N* = 106); homologous (*N* = 110)	Heterologous (47.2%); homologous (52.7 %)	63.4 (IQR: 47.3–76.6); 62.7 (SD: 16.4)	64.4 (IQR: 47.7–78.2); 62.6 (SD: 17.3)
Natori 2023 [34]	3	NCT05047640	USA	Aged 18 years or older and SOT recipient (minimum 1 month posttransplant)	4 weeks or more	(2× BNT162b2) + JNJ-78436735	Viral vector	BNT162b2	Heterologous (*N* = 28); homologous (*N* = 30)	Heterologous (60.7%); homologous (70%)	54.5 (IQR: 26–79)	59.5 (27–76)
Omma 2022 [30]	3	NCT04979949	Turkey	Aged 18 to 60 years and healthy	12 to 38 weeks	(2× CoronaVac) + TURKOVAC	Inactivated	CoronaVac	Heterologous (*N *= 108); homologous (*N* = 114)	Heterologous (68.5%); homologous (66.7%)	40 (SD: 8)	39 (SD: 9.0)
Poh 2022 [49]	3	NCT05142319	Singapore	Aged 21 years or older	30 to 36 weeks	(2× BNT162b2) + mRNA-1273	mRNA	BNT162b2	Heterologous (*N* = 49); homologous (*N* = 51)	Heterologous (44%); homologous (93.7%)	NA	NA
Reindl-Schwaighofer 2022 [35]	3	EudraCT: 2021-002927-39	Austria	Aged 18 years or older and recipient of a kidney transplantation	12 weeks	(2× mRNA-1273 or BNT162b2) + Ad26COVS1^a^	Viral vector	3× mRNA-1273 OR BNT162b2	Heterologous (*N* = 100); homologous (*N* = 101)	Heterologous (59%); homologous (58%)	61.2 (SD: 11.8)	61.2 (SD: 13.1)
Roa 2023a [47]	3	NCT05188677	Philippines	Aged 18 years or older and healthy	38 weeks	(2× Coronavac) + SCB-2019	Protein subunit	CoronaVac	Heterologous (*N* = 214; homologous (*N* = 216)	Heterologous (43.9%); homologous (35.2%)	33 (IQR: 18–72); 35 (SD: 11.9)	34 (IQR: 18–71); 35.5 (SD: 11.4)
Roa 2023b [47]	4	NCT05188677	Philippines	Aged 18 years or older and healthy	29 weeks	(3× CoronaVac) + SCB-2019	Protein subunit	CoronaVac	Heterologous (*N* = 175; homologous (*N* = 125)	Heterologous (48.8%); homologous (50.4%)	42 (IQR: 19–68); 40 (SD: 12.7)	39 (IQR: 19–72); 40.0 (SD: 12.7)
Rose 2023a [41]	3	ISRCTN (CTRI/2021/08/035648)	India	Aged 18 years or older and healthy	27 weeks	(2× COVISHIELD™) + COVAXIN®	Inactivated	COVISHIELD™	Heterologous (*N* = 99); homologous (*N* = 101)	Heterologous (60.6%); homologous (64.4%)	43.6 (SD: 13.0)	39.3 (SD: 11.3)
Rose 2023b [41]	3	ISRCTN (CTRI/2021/08/035648)	India	Aged 18 years or older and healthy	30 weeks	(2× COVAXIN®) + COVISHIELD™	Viral vector	COVAXIN®	Heterologous (*N* = 102); homologous (*N* = 102)	Heterologous (51.0%); homologous (61.8%)	43.2 (SD: 14.7)	44.4 (SD: 13.6)
Shinkai 2022 [42]	3	jRCT2031210470	Japan	Aged 20 years or older and healthy	26 weeks or more	(2× BNT162b2) + S-268019-b	Protein subunit	BNT162b2	Heterologous (*N* = 101); homologous (*N* = 103)	Heterologous (70.3%); homologous (70.6%)	30 (IQR: 21–59)	31.5 (IQR: 21–60)
Tang 2023 [55]	4	NCT05303584	China	Aged 18 years or older and healthy	28 weeks	(3× CoronaVac) + Ad5-nCov	Viral vector	CoronaVac	Heterologous (*N* = 120; homologous (*N* = 119)	Heterologous (40%); homologous (44%)	46.4 (SD: 11.2)	43.5 (SD: 12.2)
Toback 2023 [54]	4	NCT05249816	UAE	Aged 18 years or older and healthy	13 weeks or more	(3× BBIBP-CorV) + NVX-CoV2373^b^	Protein subunit	BBIBP-CorV	Heterologous (*N* = 499); homologous (*N* = 501)	Heterologous (84.8%); homologous (83.4%)	37.8 (SD: 9.0)	37.5 (SD: 8.9)
Yong 2023 [31]	3	NCT05928455	China	Aged 18–59 years and healthy	24 weeks or more	(2× CoronaVac ) + LYB001	Virus-like particle (VLP)	CoronaVac	Heterologous (*N* = 30); homologous (*N* = 29)	Heterologous (66.67%); homologous (65.52%)	27.5 (IQR: 18–54); 29.6 (SD: 7.8)	27.0 (IQR: 19–54); 30.1 (SD: 9.1)
Zhang 2022a [28]	3	ChiCTR2200057758	China	Aged 18 to 59 years and healthy	27 weeks	(2× CoronaVac) + ChAdTS-S	Viral vector	CoronaVac	Heterologous (*N* = 50); homologous (*N* = 50)	Heterologous: (50%); homologous (54.3%)	27.0 (IQR: 23.8–31.3); 28.5 (SD: 6.1)	29.0 (IQR: 25.0–34.0); 29.8 (SD: 6.1)
Zhang 2022b [28]	3	ChiCTR2200057758	China	Aged 18 to 59 years and healthy	28 weeks	(2× CoronaVac) + RQ3013	mRNA	CoronaVac	Heterologous (*N* = 50); homologous (*N* = 50)	Heterologous (44.7%); homologous (54.3%)	27.0 (IQR: 25.0–33.0); 29.0 (SD: 6.0)	29.0 (IQR: 25.0–34.0); 29.8 (SD: 6.1)
Zhang 2022c [28]	3	ChiCTR2200057758	China	Aged 18 to 59 years and healthy	27 weeks	(2× CoronaVac) + ZR202-CoV	Protein subunit	CoronaVac	Heterologous (*N* = 50); homologous (*N* = 50)	Heterologous: (42.6%); homologous: (54.3%)	28.0 (IQR: 24.0, 35.0); 29.7 (SD: 6.4)	29.0 (IQR: 25.0–34.0); 29.8 (SD:61)

^a^Contingent upon initial vaccination compound

^b^813 participants had three doses prior, and 185 participants had two doses prior. Two participants were missing

### Primary outcomes

#### All-cause mortality

**Fig. 1:**
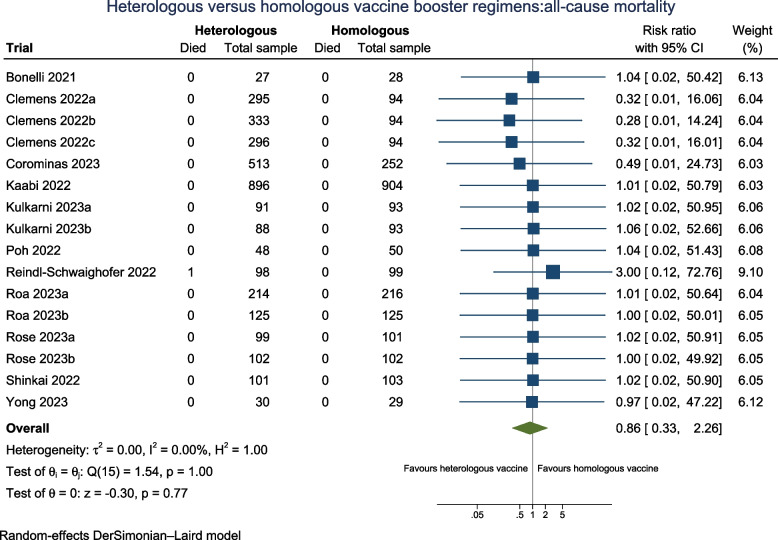
Heterologous versus homologous vaccine booster regimens: all-cause mortality

The 11 trials (*N* = 5883) which reported on all-cause mortality observed one death in an immunocompromised participant in the heterologous group because of a SAE (myocardial infarction) (Fig. 1). Five trials (45%) were assessed as having some concerns regarding bias (Additional file: FigS 24) and 5 trials (45%) followed participants 90 days or more (Additional file: FigS 20).

The meta-analysis suggested that the heterologous booster vaccines may have no effect on reducing all-cause mortality compared with homologous booster vaccines (RR 0.86; 95% CI 0.33 to 2.26; *I*^2^ 0.0%; very low certainty evidence), with comparable fixed-model and Peto OR effect estimates (Additional file: Table S3).

The trial sequential analysis (Additional file: FigS1) showed that the cumulative *Z*-curve did not cross the conventional boundaries after inclusion of eleven trials, nor reached the futility boundaries, indicating a need for more trials. It is very uncertain that subgroup analyses across heterologous booster vaccine platforms (Additional file: FigS12), number of doses (Additional file: FigS17), follow-up time (Additional file: FigS20), risk of bias (Additional file: FigS24), health status (Additional file: FigS27), and trials published before and in 2023 (Additional file: FigS31) have no effect in reducing all-cause mortality.

#### Laboratory-confirmed symptomatic COVID-19

**Fig. 2:**
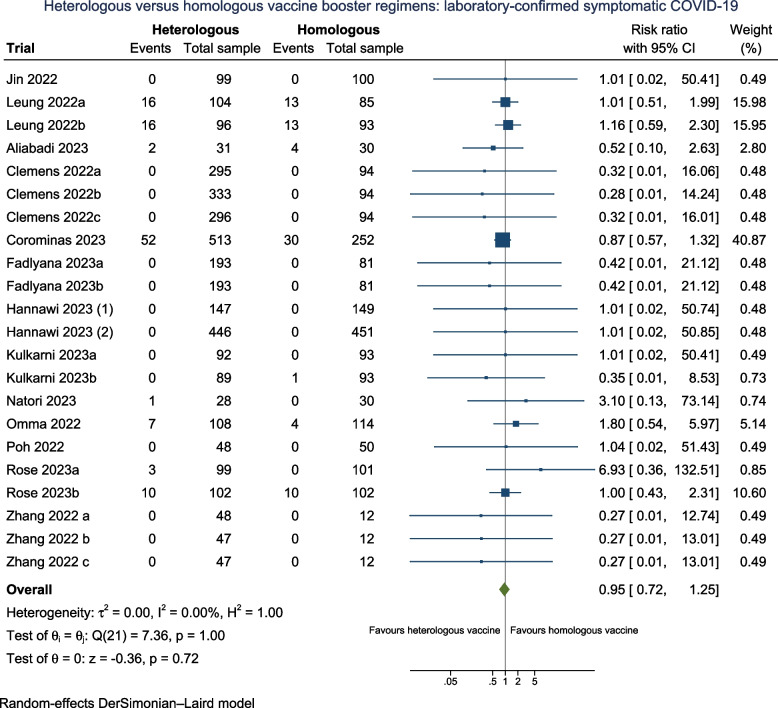
Heterologous versus homologous vaccine booster regimens: laboratory-confirmed symptomatic COVID-19

All trials either used reverse transcription polymerase chain reaction (RT-PCR) or similar laboratory tests for COVID-19 exclusively for those reporting symptoms. Thus, we were only able to report on symptomatic participants of COVID-19 and not all participants with confirmed COVID-19 as stated in our protocol. Fourteen trials (*N* = 5677) reported on symptomatic COVID-19 with 13 trials (Fig. 2) assessed as having some concerns for Domain 4 (measurement of the outcome) and one being downgraded to high risk of bias due to three domains being attributed some concerns. Seven trials (50%) followed participants 90 days or more (Additional file: FigS21). The pooled RR suggested that the heterologous booster vaccines may not have effect on risk of confirmed symptomatic COVID-19 compared with homologous booster vaccines (RR 0.95; 95% CI 0.72 to 1.25; *I*^2^ 0.0%; very low certainty evidence), which was further supported by estimates from the fixed-effect model and the Peto OR (Additional file: Table S3). The TSA showed that the cumulative *Z*-curve did not cross the conventional boundaries after inclusion of the fourteen trials, nor reached the futility boundaries, indicating a need for more trials (Additional file: FigS2).

As authors did not report the methodology of how symptomatic COVID-19 participants were diagnosed, this was reflected by assigning some concerns in Domain 4 (measurement of the outcome), therefore precluding us from performing a subgroup analysis by risk of bias. It is uncertain that subgroup analyses according to heterologous booster vaccine platforms (Additional file: Fig S13), variations in follow-up duration (Additional file: Fig S21), health status (Additional file: Fig S28), by pre-2023 and in 2023 (Additional file: Fig S32), and according to vaccine booster valency (Additional file: Fig S34), have no effect in reducing laboratory-confirmed symptomatic COVID-19 events between the two intervention groups.

#### Laboratory-confirmed severe COVID-19

**Fig. 3:**
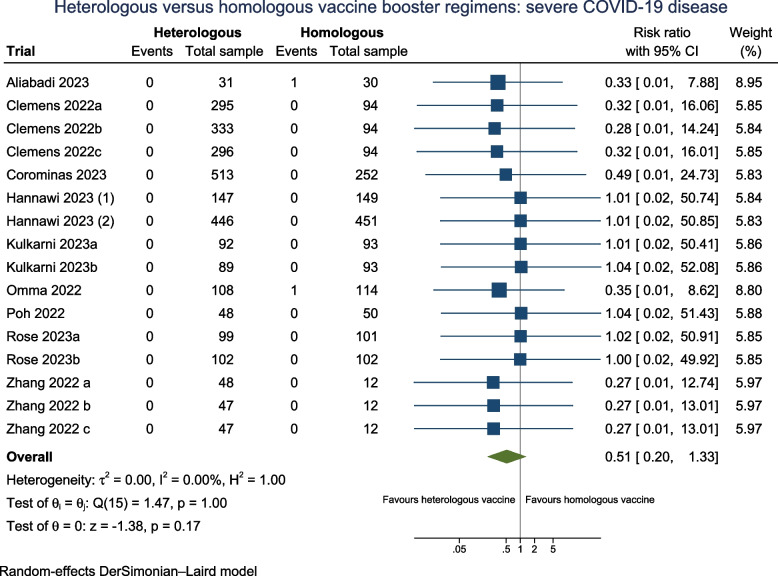
Heterologous versus homologous vaccine booster regimens: severe COVID-19 disease

Ten trials (*N* = 4494) assessed severe disease associated with laboratory-confirmed COVID-19 (Fig. 3), with all trials having some concerns for Domain 4 (measurement of the outcome). Only two participants with severe COVID-19 were reported, which occurred in the homologous booster group. Six trials (60%) followed participants 90 days or more **(**Additional file: Fig S22).

The pooled random-effects model estimates that heterologous booster doses may have no effect on reducing severe COVID-19 symptoms versus homologous booster doses (RR 0.51; 95% CI 0.20 to 1.33; *I*^2^ 0.0%; very low certainty), with comparable estimates from the fixed-effect model and Peto OR (Additional file: Table S3). The TSA underscored that the required meta-analytic sample size has not been met, thereby preventing the establishment of conclusive evidence (Additional file: FigS3). Therefore, additional trials are imperative to substantiate the impact of a heterologous vaccine regimen on laboratory-confirmed severe COVID-19 participants.

As trial authors did not report the methodology of how severe COVID participants were diagnosed, all trials measuring this outcome were assessed as having some concerns for Domain 4 (measurement of the outcome), therefore precluding us from performing a subgroup analysis by risk of bias. It is very uncertain that subgroup analyses across heterologous booster vaccine platforms (Additional file: FigS14), variations in follow-up duration (Additional file: FigS22), pre-2023 and in 2023 (Additional file: FigS33), and according to vaccine booster valency (Additional file: FigS35) have any effect in reducing laboratory-confirmed severe COVID-19 between the subgroups.

#### Serious adverse events

**Fig. 4:**
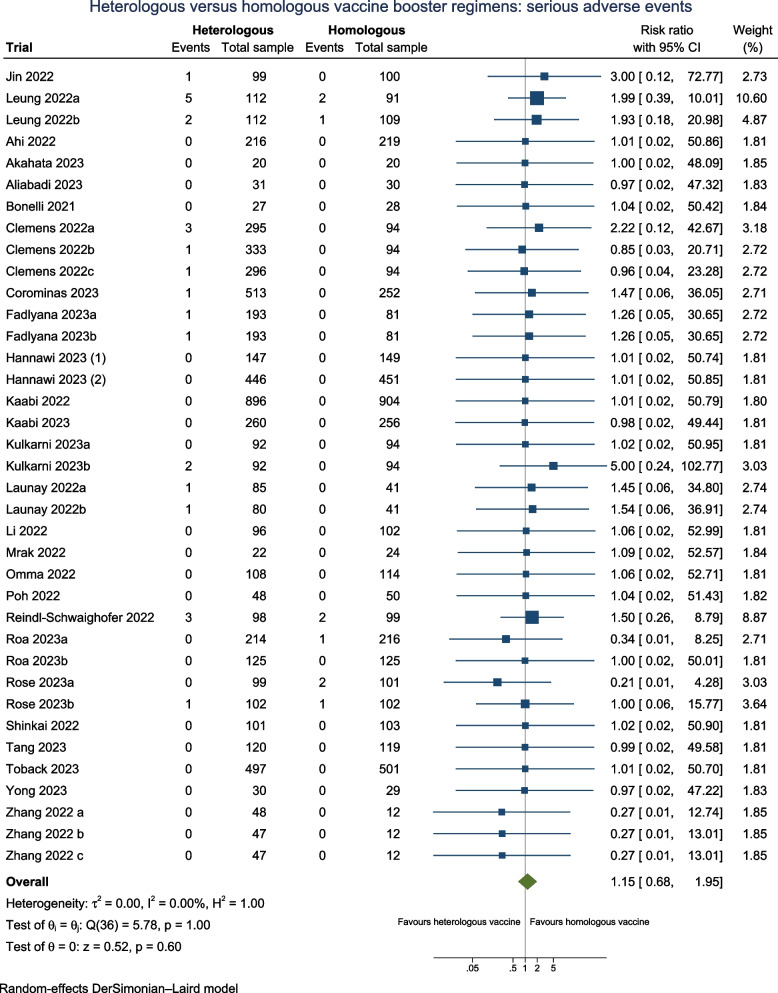
Heterologous versus homologous vaccine booster regimens: serious adverse events

Twenty-seven trials (*N* = 11,384) reported serious adverse events (SAE) when assessing the safety profile of the heterologous versus homologous booster vaccines (Fig. 4), of which 13 of trials (48%) were assessed as having one or more concerns across domains of which three trials at high risk of bias. Fourteen trials (52%) followed participants 90 days or longer**.**

The overall estimates suggest that there may be no difference on the risk for serious adverse events between heterologous booster vaccines versus homologous booster vaccines (RR 1.15; 95% CI 0.68 to 1.95; *I*^2^ 0.0%; very low certainty evidence), with comparable estimates from the fixed-effect model and Peto OR (Additional file: Table S3). The TSA reveals that the cumulative number of participants remains suboptimal, indicating the insufficiency of the accrued sample size (Additional file: FigS4). Therefore, additional trials are necessary to ascertain the impact of a heterologous vaccine regimen on serious adverse events. It is very uncertain that subgroup analyses across heterologous booster vaccine platforms (Additional file: FigS15), different doses (Additional file: FigS18), variations in follow-up duration (Additional file: FigS23), risk of bias (Additional file: FigS25), health status (Additional file: FigS29), and according to vaccine booster valency (Additional file: FigS36) may have any effect on SAE between the subgroups.

### Secondary outcomes

#### Quality of life

None of the included trials reported on health-related QoL.

#### Adverse events considered not serious

**Fig. 5:**
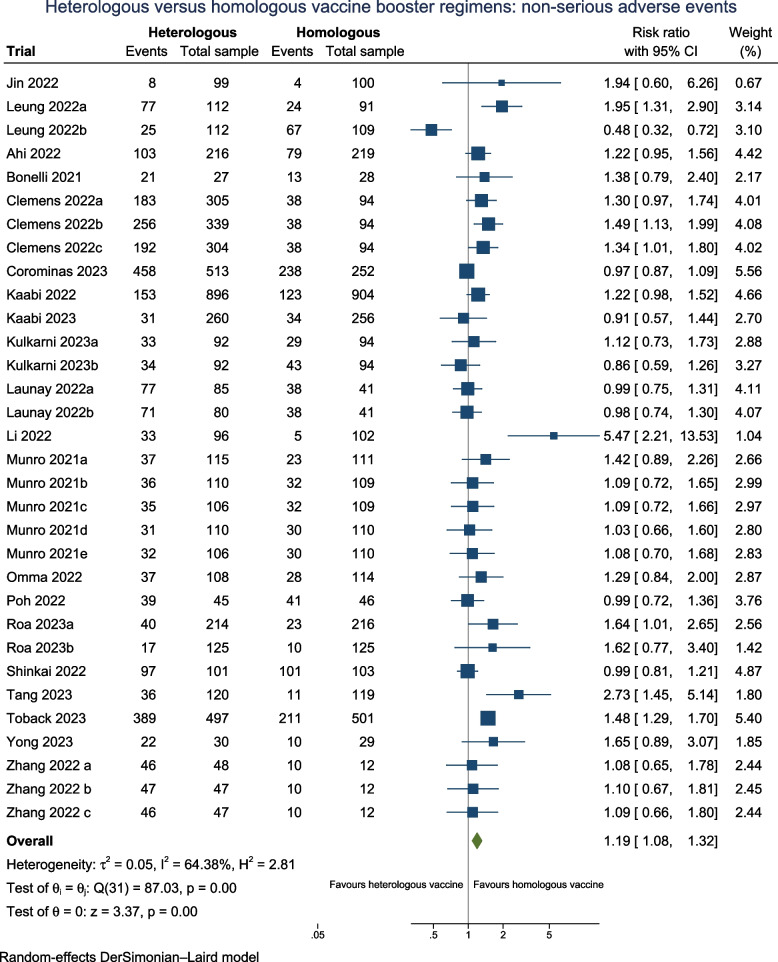
Heterologous versus homologous vaccine booster regimens: non-serious adverse events

Twenty trials (*N* = 10,008) reported on AE considered non-serious when assessing the safety profile for booster vaccines (Fig. 5), of which ten trials (50%) were considered as having one or more concerns across domains of which two were at high risks of bias. Follow-up for all trials was less than 90 days.

Most common types of AE considered non serious were fatigue, fever, injection site pain, redness, muscle pain, and headache. The overall pooled RR suggested that there may be a higher risk of AE considered non-serious by 21% in the heterologous vaccination group versus the homologous vaccination group (RR 1.19; 95% CI 1.08 to 1.32; I^2^ 64.4%; very low certainty), with concurring estimates with the fixed-effect model and Peto OR (Additional file: Table S3). The TSA showed that the cumulative *Z*-curve did not intersect the threshold indicating potential harm nor potential benefit associated with heterologous vaccines after incorporating the 20 trials (Additional file: FigS5).

Subgroup analyses based on different doses (Additional file: Fig S19), risk of bias (Additional file: Fig S26), and health status (Additional file: Fig S30) did not impact the pooled relative risk (RR) or reduce heterogeneity. The lack of difference in effect due to different doses on adverse events (AEs) considered non-serious remains very uncertain across subgroups. Furthermore, the evidence for differential higher risks of non-serious AE with protein-based vaccine boosters, viral-vectored booster platforms, and mRNA vaccine booster platforms remain very uncertain due to an even higher risk of imprecision (RR 1.13; 95% CI 1.00 to 1.29; *I*^2^: 62.5%), (RR 1.51; 95% CI 1.16 to 1.97; *I*^2^: 56.2%,) and (RR 1.25; 95% CI 1.00 to 1.56), respectively (Additional file: FigS16).

#### Publication bias

No asymmetry for all-cause mortality, symptomatic COVID-19, severe COVID-19, and SAE (Additional file: Fig S37-40) were observed in the funnel plots, providing evidence against publication bias, which was further corroborated by Egger’s tests showing no significant evidence of publication bias. For adverse events considered non-serious, despite the presence of slight asymmetry in the funnel plot for the outcome (Additional file: FigS44), the significant result from the Egger’s test (*P*: 0.02) suggests evidence of publication bias for non-serious adverse events. It is noteworthy that substantial heterogeneity among the included trials could potentially account for the observed asymmetry, introducing some uncertainty into our findings.

## Discussion

In this updated living vaccine project valid until the end of 2023, we focused on gathering evidence from 29 trials comparing heterologous-based booster versus homologous-based booster regimens, of which two compared multivalent versus bivalent boosters. We found no evidence of different effects on mortality, laboratory-confirmed symptomatic COVID-19, laboratory-confirmed severe COVID-19, or SAE. Our TSAs revealed that the accrued sample size was suboptimal to make any robust conclusions of any difference of effects on these outcomes. We found no data on QoL. Nevertheless, we found that heterologous booster regimens may increase the occurrence of AE considered non-serious, but more data will be required to confirm this finding.

Heterogeneity was only encountered assessing AE considered non-serious. Notably, for this outcome, subgroup analyses across vaccine platforms, doses, risk of bias, and health status of participants did not reduce the high level of heterogeneity, which remained above 50%. Due to limited sample sizes, we cannot confidently determine significant differences or lack thereof for all outcomes.

Thus, at this juncture, the very low certainty of evidence yielded from this systematic review does not allow an assessment of beneficial and harmful effects of combining the two different types of vaccine platform, thereby providing limited evidence supporting any firm conclusions. Thus, it would be premature to infer whether lack of statistical significance is due to insufficient sample size or due to no differences between heterologous and homologous booster regimens.

To our knowledge, no other systematic review comprising only randomised clinical trials exists, thus hindering direct comparisons to be made. Three meta-analyses were published between April and August 2022, with the bulk of evidence emanating from observational studies [5–7]. Deng et al. [6] reported higher vaccine effectiveness for symptomatic COVID-19 and severe symptoms associated with COVID-19 with heterologous boosters (56.8% compared to 17.3% and 97.4% compared to 93.4%, respectively) [6]. Conversely, Au et al. (2022) found comparable effectiveness between heterologous and homologous three-dose regimens in preventing COVID-19 symptomatic and severe infections [7]. Regarding safety outcomes, our findings align with Deng et al. [6], who reported higher odds for adverse events considered non-serious in the heterologous booster group, in disagreement with Cheng et al. [5] who reported a higher incidence of total adverse events in the homologous group booster group [5]. However, these discrepancies may be attributed to confounding factors, including location-based differences in vaccination strategies.

## Strengths and limitations

Strengths related to our methodology include the use of five biomedical databases drawing from a combination of approaches to increase the likelihood of capturing all eligible trials. Second, we only included randomised clinical trials. Third, we employed our general search strategy as defined by the protocol followed by a specific search strategy tailored to our specific research question, which was later complemented with the use of the snowballing method. Fourth, we conducted TSAs to control type I and type II errors and strengthen our assessment of the imprecision domain in GRADE.

Our eligible trials have several strengths. Firstly, the inclusion of participants from diverse geographical regions supports the generalisation of results, increasing the applicability of our findings to broader populations. Furthermore, by utilising various vaccine regimen combinations in the heterologous arm, compared with different homologous vaccine regimens, we further enhance the generalisation of our results in addressing our broad research question, whether heterologous regimens are more likely to improve vaccine efficacy and safety.

However, interpretation of our findings warrants caution and cognisance of certain methodological limitations, as reflected in the very low certainty we have in the evidence, largely attributable to the non-negligible percentage of RCT not being free of potential biases, imprecision, and heterogeneity. Secondly, we were unable to adequately assess the quality of RCT reporting on vaccine efficiency as none of the eligible trials reporting on these outcomes described the methodology for assessing this efficiency. In addition, whilst including trials from different geographical regions with varying patterns of sublineage predominance, vaccination combinations, and intervals between prime and boost doses using different vaccine regimens may help generalise findings, this diversity may also lead to residual heterogeneity, as seen in the case of adverse events considered non-serious.

Whilst our study provides valuable insights into the efficacy and safety outcomes of homologous compared with heterologous vaccine regimens across various vaccine platforms, we acknowledge that the absence of trials involving recombinant protein boosters may have limited our exploration of the effect of protein-based heterologous boosters. Additionally, the majority of the trials had a follow-up time of less than 3 months, along with large inoculation time intervals between doses, potentially resulting in failure to adequately gauge benefits and harms. The absence of disaggregated data for older adults, who along with the immunocompromised population, are poised to benefit the most from a booster dose, further limits our analyses.

Hence, this systematic review underscores the imperative for more robust randomised clinical trials to corroborate either all non-significant differences observed or explore the possibility of a differential effect between heterologous versus homologous booster regimen, also among older adults.

## Conclusions

Our living systematic review provides current insights into the comparative efficacy and safety of heterologous versus homologous COVID-19 booster regimens. Upon evaluating three vaccine efficacy outcomes, i.e., all-cause mortality, symptomatic COVID-19, and severe COVID-19, no adequate accrued sample size was reached to be able to conclude a lack of difference in prevention between the heterologous versus homologous booster vaccine regimens. In terms of safety outcomes, whilst heterologous vaccine regimens may lead to higher occurrences of AE considered non-serious in contrast to SAE which showed a pooled relative risk range that encompassed the line of no effect, our TSAs pointed to inadequate sample size for both outcomes. As multivalent vaccine heterologous boosters become more prominent, future randomised clinical trials should prioritise diverse populations, including older adults and immunocompromised people and ensure standardised assessment to optimise vaccination strategies and global pandemic control efforts.

### Supplementary Information

Additional file 1: Tables S1–S2 and Figures S1–43.

## Data Availability

The datasets used and/or analysed during the current study are available from the corresponding author on reasonable request.

## Abbreviations

AE: Adverse events
CI: Confidence intervals
COVID-19: Coronavirus disease 2019
GRADE: Grading of Recommendations, Assessment, Development, and Evaluation
mRNA: Messenger RNA
OR: Odds ratio
QoL: Quality of life
RCT: Randomised clinical trials
RR: Relative risk or risk ratio
RT-PCR: Reverse transcription polymerase chain reaction
SAE: Serious adverse events
SARS-CoV-2: Severe respiratory syndrome coronavirus 2
TSA: Trial sequential analysis
VOIs: Variants of interest
VUMs: Variants under monitoring
WHO: World Health Organization
