# Supplementary material for: Heterologous versus homologous COVID-19 booster vaccinations for adults: systematic review with meta-analysis and trial sequential analysis of randomised clinical trials
Source: BMC Med. 2024 Jun 24;22:263. doi: 10.1186/s12916-024-03471-3 (PMC11197367; doi:10.1186/s12916-024-03471-3)
Supplement: Supplementary file 1 — Additional file 1: Tables S1–S2 and Figures S1–43. [file 12916_2024_3471_MOESM1_ESM.docx]

**­­­Additional file**

**­**

# **Heterologous versus Homologous COVID-19 Booster Vaccinations for Adults: Systematic Review with Meta-Analysis and Trial Sequential Analysis of Randomised Clinical trials**

By

Mark Aninakwah Asante*, Martin Ekholm Michelsen*, Mithuna Mille Balakumar, Buddheera Kumburegama, Amin Sharifan, Allan Randrup Thomsen, Steven Kwasi Korang, Christian Gluud, Sonia Menon

**Shared first authors***

**Corresponding author # Dr Sonia Menon**

Contents

[**Extended method section**](#_heading=h.30j0zll) 3

[**Search Strategies**](#_heading=h.tyjcwt) 6

[**Sensitivity analysis** 1](#_heading=h.1t3h5sf)0

[**Trial Sequential Analysis (TSA)** 1](#_heading=h.3rdcrjn)0

[**Risk of bias assessment** 1](#_heading=h.35nkun2)8

[**Additional supplementary figures** 2](#_heading=h.1y810tw)2

[**Publication**](#_heading=h.3rdcrjn) **bias** [1](#_heading=h.3rdcrjn)0

[**PRISMA**](#_heading=h.35nkun2) **flow chart** [1](#_heading=h.35nkun2)8

[**PRISMA**](#_heading=h.1y810tw) **checklist** [2](#_heading=h.1y810tw)2

#

## Extended method section

### Databases

We searched the following databases: Cochrane Central Register of Controlled Trials, Medical Literature Analysis and Retrieval System Online (MEDLINE Ovid), Excerpta Medica database (Embase Ovid), Latin American and Caribbean Health Sciences Literature (LILACS; Bireme), Science Citation Index Expanded (SCI-EXPANDED; Web of Science), Conference Proceedings Citation Index—Science (CPCI-S; Web of Science), Chinese Biomedical Literature Database (CBM), China Network Knowledge Information (CNKI), Chinese Science Journal Database (VIP), and Wanfang Database to identify relevant trials. We searched all databases from their inception to the present. We also searched special COVID-19 trial sites and preprint servers for health sciences. We also searched online trial registries such as ClinicalTrials.gov (clinicaltrials.gov), the Chinese Clinical Trial Registry (www.chictr.org.cn), the European Medicines Agency (EMA) (www.ema.europa.eu/), the World Health Organization (WHO) International Clinical Trials Registry Platform (www.who.int/ictrp/), and the Food and Drug Administration (FDA) (www.fda.gov/) for ongoing or unpublished trials. We searched for grey literature in the System for Information on Grey Literature in Europe OpenGrey (www.opengrey.eu). For detailed search strategies for all electronic searches, see **Search Strategies** below.

No restrictions were imposed regarding publication status, publication year, and language.

We included randomised clinical trials with participants irrespective of prior exposure, age, sex, comorbidities, immune status, and risk group. Any booster vaccine aiming to prevent COVID-19 irrespective of dose and duration of administration was eligible. Randomised clinical trials with homologous control group, aiming at preventing COVID-19, were eligible.

### Data collection

In this living systematic review, two independent investigators (SM, MAA) received an updated literature search file and included relevant newly published or unpublished trials every two weeks. A detailed description of the living systematic review methodology can be found in our protocol.

Four authors working in pairs (MAA, MEM, MMB, BW, MBK, AS) independently extracted data from included trials in a predefined form. All the extracted data were double-checked by a third author (MEM, MAA, SM, AS). Disagreements were resolved by discussion or, if required, through consultation with a fourth author (MAA or SM). We contacted the trial authors by email to specify any missing data, which may not be reported sufficiently or not at all in the publication.

### Definition of outcomes

| **Primary outcomes** | **Definition of outcome** |
| --- | --- |
| All-cause mortality | Death of all causes |
| Vaccine efficacy | Either preventing COVID-19 symptoms plus positive laboratory test, preventing severe COVID-19 symptoms plus positive laboratory test, or preventing positive laboratory test only; and serious adverse events. |
| Serious adverse events | Proportion of participants with one or more serious adverse events. We will use the International Conference on Harmonization of technical requirements for registration of pharmaceuticals for human use—Good Clinical Practice (ICH-GCP) definition of a serious adverse event, which is any untoward medical occurrence that resulted in death, was life-threatening, required hospitalization or prolonging of existing hospitalization, and resulted in persistent or significant disability or jeopardized the participant. If the trialists do not use the ICH-GCP definition, we will include the data if the trialists use the term “serious adverse event.” If the trialists do not use the ICH-GCP definition nor use the term serious adverse event, then we will also include the data if the event clearly fulfills the ICH-GCP definition for a serious adverse event. |
| **Secondary outcomes** |  |
| Health-related quality of life | Health-related quality of life (assessed on any valid continuous scale) |
| Adverse events not considered serious | Proportion of participants with one or more adverse events not considered serious. We will exploratory assess each type of adverse events not considered serious separately (see below) |

*Table S1: Definition of outcomes*

### Search Strategies

**Cochrane Central Register of Controlled Trials (2023, Issue 7) in the Cochrane Library ((4978 hits)**

#1 MeSH descriptor: [Coronavirus Infections] explode all trees

#2 ((corona near (virus or viral or infection*)) or coronaviri* or covid* or sars-cov-2)

#3 #1 or #2

#4 MeSH descriptor: [Vaccines] explode all trees

#5 MeSH descriptor: [Vaccination] explode all trees

#6 MeSH descriptor: [Immunization] explode all trees

#7 (vaccin* or immun*)

#8 ((RNA or DNA or recombinant* or vector* or inactivat* or live attenuat* or dendritic or adeno* or encapsulated or plasmid* or protein subunit or peptid* or spike) near/5 vaccin*)

#9 (BNT162 or AZD1222 or Ad5* or INO-4800 or bacTRL* or PRO-nCov-100* or v-sars or vero cell* or SCB-2019 or NVX-COV2373 or BCG vaccin* or VPM1002 or aAPC or AV-COVID-19 or TBD* or Ad26* or MVA* or hAd5* or PiCoVacc or BBIBP-CorV)

#10 #4 or #5 or #6 or #7 or #8 or #9

#11 #3 and #10

**MEDLINE Ovid (1946 to 21 December 2023) ((7003 hits)**

1. exp Coronavirus Infections/

2. ((corona adj (virus or viral or infection*)) or coronaviri* or covid* or sars-cov-2).mp. [mp=title, abstract, original title, name of substance word, subject heading word, floating sub-heading word, keyword heading word, organism supplementary concept word, protocol supplementary concept word, rare disease supplementary concept word, unique identifier, synonyms]

3. 1 or 2

4. exp Vaccines/

5. exp Vaccination/

6. exp Immunization/

7. (vaccin* or immun*).mp. [mp=title, abstract, original title, name of substance word, subject heading word, floating sub-heading word, keyword heading word, organism supplementary concept word, protocol supplementary concept word, rare disease supplementary concept word, unique identifier, synonyms]

8. ((RNA or DNA or recombinant* or vector* or inactivat* or live attenuat* or dendritic or adeno* or encapsulated or plasmid* or protein subunit or peptid* or spike) adj5 vaccin*).mp. [mp=title, abstract, original title, name of substance word, subject heading word, floating sub-heading word, keyword heading word, organism supplementary concept word, protocol supplementary concept word, rare disease supplementary concept word, unique identifier, synonyms]

9. (BNT162 or AZD1222 or Ad5* or INO-4800 or bacTRL* or PRO-nCov-100* or v-sars or vero cell* or SCB-2019 or NVX-COV2373 or BCG vaccin* or VPM1002 or aAPC or AV-COVID-19 or TBD* or Ad26* or MVA* or hAd5* or PiCoVacc or BBIBP-CorV).mp. [mp=title, abstract, original title, name of substance word, subject heading word, floating sub-heading word, keyword heading word, organism supplementary concept word, protocol supplementary concept word, rare disease supplementary concept word, unique identifier, synonyms]

10. 4 or 5 or 6 or 7 or 8

11. 3 and 10

12. (randomized controlled trial or controlled clinical trial).pt. or clinical trials as topic.sh. or trial.ti.

13. (random* or blind* or placebo* or meta-analys*).mp. [mp=title, abstract, original title, name of substance word, subject heading word, floating sub-heading word, keyword heading word, organism supplementary concept word, protocol supplementary concept word, rare disease supplementary concept word, unique identifier, synonyms]

14. 11 and (12 or 13)

**Embase Ovid (1974 to December 21, 2023) (11713 hits)**

1. exp coronaviridae/

2. ((corona adj (virus or viral or infection*)) or coronaviri* or covid* or sars-cov-2).mp. [mp=title, abstract, heading word, drug trade name, original title, device manufacturer, drug manufacturer, device trade name, keyword, floating subheading word, candidate term word]

3. 1 or 2

4. exp vaccine/

5. exp vaccination/

6. exp immunization/

7. (vaccin* or immun*).mp. [mp=title, abstract, heading word, drug trade name, original title, device manufacturer, drug manufacturer, device trade name, keyword, floating subheading word, candidate term word]

8. ((RNA or DNA or recombinant* or vector* or inactivat* or live attenuat* or dendritic or adeno* or encapsulated or plasmid* or protein subunit or peptid* or spike) adj5 vaccin*).mp. [mp=title, abstract, heading word, drug trade name, original title, device manufacturer, drug manufacturer, device trade name, keyword, floating subheading word, candidate term word]

9. (BNT162 or AZD1222 or Ad5* or INO-4800 or bacTRL* or PRO-nCov-100* or v-sars or vero cell* or SCB-2019 or NVX-COV2373 or BCG vaccin* or VPM1002 or aAPC or AV-COVID-19 or TBD* or Ad26* or MVA* or hAd5* or PiCoVacc or BBIBP-CorV).mp. [mp=title, abstract, heading word, drug trade name, original title, device manufacturer, drug manufacturer, device trade name, keyword, floating subheading word, candidate term word]

10. 4 or 5 or 6 or 7 or 8 or 9

11. 3 and 10

12. exp randomized controlled trial/

13. exp controlled clinical trial/

14. exp intermethod comparison/

15. exp double blind procedure/

16. (random* or blind* or placebo* or meta-analys*).mp. [mp=title, abstract, heading word, drug trade name, original title, device manufacturer, drug manufacturer, device trade name, keyword, floating subheading word, candidate term word]

17. 12 or 13 or 14 or 15 or 16

18. 11 and 17

**LILACS (Bireme; 1982 to December 21 2023) ((2531 hits))**

((corona and (virus or viral or infection$)) or coronaviri$ or covid$ or sars-cov-2) [Words] and (vaccin$ or immun$) or ((RNA or DNA or recombinant$ or vector$ or inactivat$ or live attenuat$ or dendritic or adeno$ or encapsulated or plasmid$ or protein subunit or peptid$ or spike) and vaccin$) or (BNT162 or AZD1222 or Ad5$ or INO-4800 or bacTRL$ or PRO-nCov-100$ or v-sars or vero cell$ or SCB-2019 or NVX-COV2373 or BCG vaccin$ or VPM1002 or aAPC or AV-COVID-19 or TBD$ or Ad26$ or MVA$ or hAd5$ or PiCoVacc or BBIBP-CorV) [Words]

**BIOSIS (Web of Science; 1969 to December 21 2023) (3798 hits)**

#8 #7 AND #6

#7 TI=(random* or blind* or placebo* or meta-analys* or trial*) OR TS=(random* or blind* or placebo* or meta-analys*)

#6 #5 AND #1

#5 #4 OR #3 OR #2

#4 TS=(BNT162 or AZD1222 or Ad5* or INO-4800 or bacTRL* or PRO-nCov-100* or v-sars or vero cell* or SCB-2019 or NVX-COV2373 or BCG vaccin* or VPM1002 or aAPC or AV-COVID-19 or TBD* or Ad26* or MVA* or hAd5* or PiCoVacc or BBIBP-CorV)

#3 TS=((RNA or DNA or recombinant* or vector* or inactivat* or live attenuat* or dendritic or adeno* or encapsulated or plasmid* or protein subunit or peptid* or spike) and vaccin*)

#2 TS=(vaccin* or immun*)

#1 TS=((corona near (virus or viral or infection*)) or coronaviri* or covid* or sars-cov-2)

**Science Citation Index Expanded (1900 to 20 July 2023) and Conference Proceedings Citation Index – Science (1990 to December 21 2023) (Web of Science) (5516 hits)**

#8 #7 AND #6

#7 TI=(random* or blind* or placebo* or meta-analys* or trial*) OR TS=(random* or blind* or placebo* or meta-analys*)

#6 #5 AND #1

#5 #4 OR #3 OR #2

#4 TS=(BNT162 or AZD1222 or Ad5* or INO-4800 or bacTRL* or PRO-nCov-100* or v-sars or vero cell* or SCB-2019 or NVX-COV2373 or BCG vaccin* or VPM1002 or aAPC or AV-COVID-19 or TBD* or Ad26* or MVA* or hAd5* or PiCoVacc or BBIBP-CorV)

#3 TS=((RNA or DNA or recombinant* or vector* or inactivat* or live attenuat* or dendritic or adeno* or encapsulated or plasmid* or protein subunit or peptid* or spike) and vaccin*)

#2 TS=(vaccin* or immun*)

#1 TS=((corona near (virus or viral or infection*)) or coronaviri* or covid* or sars-cov-2)

#

### Additional Search strategies

Searches performed 5 April 2023

Total number of records identified: 1406 records

Number of duplicates excluded: 419 records

Number of records in final list: 987 records

Cochrane Central Register of Controlled Trials (2023, Issue 4) in the Cochrane Library (363 hits)

#1 MeSH descriptor: [Coronavirus Infections] explode all trees

#2 ((corona near (virus or viral or infection*)) or coronaviri* or covid* or sars-cov-2)

#3 #1 or #2

#4 MeSH descriptor: [Vaccines] explode all trees

#5 MeSH descriptor: [Vaccination] explode all trees

#6 MeSH descriptor: [Immunization] explode all trees

#7 (vaccin* or immun*)

#8 ((RNA or DNA or recombinant* or vector* or inactivat* or live attenuat* or dendritic or adeno* or encapsulated or plasmid* or protein subunit or peptid* or spike or "virus like particle*" or "antigen presenting cell*") near/5 vaccin*)

#9 (BNT162 or AZD1222 or Ad5* or INO-4800 or bacTRL* or PRO-nCov-100* or v-sars or vero cell* or SCB-2019 or NVX-COV2373 or BCG vaccin* or VPM1002 or aAPC or AV-COVID-19 or TBD* or Ad26* or MVA* or hAd5* or PiCoVacc or BBIBP-CorV or VVnr or VVr or VLP or BacAg-SpV)

#10 #4 or #5 or #6 or #7 or #8 or #9

#11 booster*

#12 #3 and #10 and #11

### MEDLINE Ovid (1946 to 5 April 2023) (265 hits)

1. exp Coronavirus Infections/

2. ((corona adj (virus or viral or infection*)) or coronaviri* or covid* or sars-cov-2).mp. [mp=title, book title, abstract, original title, name of substance word, subject heading word, floating sub-heading word, keyword heading word, organism supplementary concept word, protocol supplementary concept word, rare disease supplementary concept word, unique identifier, synonyms]

3. 1 or 2

4. exp Vaccines/

5. exp Vaccination/

6. exp Immunization/

7. (vaccin* or immun*).mp. [mp=title, book title, abstract, original title, name of substance word, subject heading word, floating sub-heading word, keyword heading word, organism supplementary concept word, protocol supplementary concept word, rare disease supplementary concept word, unique identifier, synonyms]

8. ((RNA or DNA or recombinant* or vector* or inactivat* or live attenuat* or dendritic or adeno* or encapsulated or plasmid* or protein subunit or peptid* or spike or "virus like particle*" or "antigen presenting cell*") adj5 vaccin*).mp. [mp=title, book title, abstract, original title, name of substance word, subject heading word, floating sub-heading word, keyword heading word, organism supplementary concept word, protocol supplementary concept word, rare disease supplementary concept word, unique identifier, synonyms]

9. (BNT162 or AZD1222 or Ad5* or INO-4800 or bacTRL* or PRO-nCov-100* or v-sars or vero cell* or SCB-2019 or NVX-COV2373 or BCG vaccin* or VPM1002 or aAPC or AV-COVID-19 or TBD* or Ad26* or MVA* or hAd5* or PiCoVacc or BBIBP-CorV or VVnr or VVr or VLP or BacAg-SpV).mp. [mp=title, book title, abstract, original title, name of substance word, subject heading word, floating sub-heading word, keyword heading word, organism supplementary concept word, protocol supplementary concept word, rare disease supplementary concept word, unique identifier, synonyms]

10. 4 or 5 or 6 or 7 or 8

11. 3 and 10

12. (randomized controlled trial or controlled clinical trial).pt. or clinical trials as topic.sh. or trial.ti.

13. (random* or blind* or placebo* or meta-analys*).mp. [mp=title, book title, abstract, original title, name of substance word, subject heading word, floating sub-heading word, keyword heading word, organism supplementary concept word, protocol supplementary concept word, rare disease supplementary concept word, unique identifier, synonyms]

14. 11 and (12 or 13)

**Embase Ovid (1974 to 5 April 2023) (398 hits)**

1. exp coronaviridae/

2. ((corona adj (virus or viral or infection*)) or coronaviri* or covid* or sars-cov-2).mp. [mp=title, abstract, heading word, drug trade name, original title, device manufacturer, drug manufacturer, device trade name, keyword heading word, floating subheading word, candidate term word]

3. 1 or 2

4. exp vaccine/

5. exp vaccination/

6. exp immunization/

7. (vaccin* or immun*).mp. [mp=title, abstract, heading word, drug trade name, original title, device manufacturer, drug manufacturer, device trade name, keyword heading word, floating subheading word, candidate term word]

8. ((RNA or DNA or recombinant* or vector* or inactivat* or live attenuat* or dendritic or adeno* or encapsulated or plasmid* or protein subunit or peptid* or spike or "virus like particle*" or "antigen presenting cell*") adj5 vaccin*).mp. [mp=title, abstract, heading word, drug trade name, original title, device manufacturer, drug manufacturer, device trade name, keyword heading word, floating subheading word, candidate term word]

9. (BNT162 or AZD1222 or Ad5* or INO-4800 or bacTRL* or PRO-nCov-100* or v-sars or vero cell* or SCB-2019 or NVX-COV2373 or BCG vaccin* or VPM1002 or aAPC or AV-COVID-19 or TBD* or Ad26* or MVA* or hAd5* or PiCoVacc or BBIBP-CorV or VVnr or VVr or VLP or BacAg-SpV).mp. [mp=title, abstract, heading word, drug trade name, original title, device manufacturer, drug manufacturer, device trade name, keyword heading word, floating subheading word, candidate term word]

10. 4 or 5 or 6 or 7 or 8 or 9

11. 3 and 10

12. exp randomized controlled trial/

13. exp controlled clinical trial/

14. exp intermethod comparison/

15. exp double blind procedure/

16. (random* or blind* or placebo* or meta-analys*).mp. [mp=title, abstract, heading word, drug trade name, original title, device manufacturer, drug manufacturer, device trade name, keyword heading word, floating subheading word, candidate term word]

17. 12 or 13 or 14 or 15 or 16

18. 11 and 17

**LILACS (VHL Regional Portal; 1982 to 5 April 2023) (31 hits)**

(((corona AND (virus OR viral OR infection*)) OR coronaviri* OR covid* OR sars-cov-2)) AND ((vaccin* OR immun*) OR ((rna OR dna OR recombinant* OR vector* OR inactivat* OR live attenuat* OR dendritic OR adeno* OR encapsulated OR plasmid* OR protein subunit OR peptid* OR spike OR virus like particle* OR antigen presenting cell*) AND vaccin*) OR (bnt162 OR azd1222 OR ad5* OR ino-4800 OR bactrl* OR pro-ncov-100* OR v-sars OR vero cell* OR scb-2019 OR nvx-cov2373 OR bcg vaccin* OR vpm1002 OR aapc OR av-covid-19 OR tbd* OR ad26* OR mva* OR had5* OR picovacc OR bbibp-corv OR vvnr OR vvr OR vlp OR bacag-spv)) AND (booster*) AND ( db:("LILACS"))

**BIOSIS (Web of Science; 1969 to 5 April 2023) (127 hits)**

#9 #7 AND #8

#8 TI=(random* or blind* or placebo* or meta-analys* or trial*) OR TS=(random* or blind* or placebo* or meta-analys*)

#7 #1 AND #5 AND #6

#6 TS=(booster*)

#5 #4 OR #3 OR #2

#4 TS=(BNT162 or AZD1222 or Ad5* or INO-4800 or bacTRL* or PRO-nCov-100* or v-sars or vero cell* or SCB-2019 or NVX-COV2373 or BCG vaccin* or VPM1002 or aAPC or AV-COVID-19 or TBD* or Ad26* or MVA* or hAd5* or PiCoVacc or BBIBP-CorV or VVnr or VVr or VLP or BacAg-SpV)

#3 TS=((RNA or DNA or recombinant* or vector* or inactivat* or live attenuat* or dendritic or adeno* or encapsulated or plasmid* or protein subunit or peptid* or spike or virus like particle* or antigen presenting cell*) and vaccin*)

#2 TS=(vaccin* or immun*)

#1 TS=((corona near (virus or viral or infection*)) or coronaviri* or covid* or sars-cov-2)

**Science Citation Index Expanded (1900 to 5 April 2023) and Conference Proceedings Citation Index – Science (1990 to 5 April 2023) (Web of Science) (222 hits)**

#9 #7 AND #8

#8 TI=(random* or blind* or placebo* or meta-analys* or trial*) OR TS=(random* or blind* or placebo* or meta-analys*)

#7 #1 AND #5 AND #6

#6 TS=(booster*)

#5 #4 OR #3 OR #2

#4 TS=(BNT162 or AZD1222 or Ad5* or INO-4800 or bacTRL* or PRO-nCov-100* or v-sars or vero cell* or SCB-2019 or NVX-COV2373 or BCG vaccin* or VPM1002 or aAPC or AV-COVID-19 or TBD* or Ad26* or MVA* or hAd5* or PiCoVacc or BBIBP-CorV or VVnr or VVr or VLP or BacAg-SpV)

#3 TS=((RNA or DNA or recombinant* or vector* or inactivat* or live attenuat* or dendritic or adeno* or encapsulated or plasmid* or protein subunit or peptid* or spike or virus like particle* or antigen presenting cell*) and vaccin*)

#2 TS=(vaccin* or immun*)

#1 TS=((corona near (virus or viral or infection*)) or coronaviri* or covid* or sars-cov-2)

### Sensitivity analysis

**Fixed-effect model and Peto odds ratio by outcome**

| **Outcome** | **Fixed-effect model** | **Peto odds ratio (OR)** |
| --- | --- | --- |
| All-cause mortality | RR 0.91; 95% CI 0.36 to 2.28 | OR 1.61; 95% CI 0.83 to 3.12 |
| Symptomatic COVID-19 | RR 0.97; 95% CI 0.74 to 1.27 | OR 1.01; 95% CI 0.74 to 1.39 |
| Severe COVID-19 | RR 0.53; 95% CI 0.21 to 1.32 | OR 0.14; 95% CI 0.00 to 2.23 |
| Serious adverse events | RR: 1.17; 95 % CI 0.71 to 1.93 | OR: 1.89; 95% CI 0.93 to 3.85 |
| Adverse events considered non serious | RR:1.19; 95%CI 1.13 to 1.26 | OR: 1.30; 95% CI 1.20 to 1.41 |

**Table S2: Fixed-effect model and Peto odds ratio by outcome**

### Trial Sequential Analysis (TSA)

We used a relative risk reduction of 20% for all dichotomous outcomes as we are comparing two active booster vaccines versus each other, an alpha of 1.67% for all our outcomes, a beta of 10%, and the observed diversity as suggested by the trials in the meta-analysis. Therefore, we did not conduct sensitivity analyses with larger intervention effects as stated in our protocol.

### Difference between protocol and review

We planned to include exploratory outcomes SARS-CoV-2 neutralising antibody titers, SARS-CoV-2, IgG-binding antibody titers, seroconversion for SARS-CoV-2 neutralising antibody, seroconversion for SARS-CoV-2 IgG-binding antibody, individual types of serious adverse events, and individual types of non-serious adverse events. These outcomes may be reported in a subsequent systematic review.

As the transitivity assumption was not upheld, which in turn prevents valid indirect comparisons between treatments, a network meta-analysis could not be performed. We did not perform Trial Sequential Analysis with a relative risk reduction (RRR) of 50% or 70% when assessing the effects of the booster vaccines, as two active vaccines were compared and such large differences seem unlikely.

**TSA for heterologous versus homologous COVID-19 vaccine regimens on all-cause mortality in healthy and immunocompromised participants**

The TSA is constructed with the x-axis denoting the accumulated number of participants and the required number of participants. The y-axis denotes the cumulative Z values in the TSA. The blue line (Z-curve) shows the cumulative Z value. The TSA showed that the diversity-adjusted required information size (DARIS) is 59,675 participants, calculated based on an assumed proportion of events of 2% in the control group; a RRR of 20%; alpha (type Ⅰ error) of 1.67%; beta (type Ⅱ error) of 10%; and diversity of 0%. The cumulative Z-curve did not cross the conventional boundary after inclusion of 11 trials (5,883 participants), nor the trial sequential monitoring boundaries of benefit nor harm demonstrating that no conclusive evidence was found. The TSA-adjusted CI was 0.02 to 43.9. The horizontal green dotted lines show the threshold for significance in conventional meta-analysis, at 1.96 and ̶ 1.96 of the Z value, corresponding to the P-value of 0.05. The red dotted lines from the left top and bottom corners show the trial sequential boundaries for benefit or harm, respectively, representing the threshold for statistical significance in the TSA. The red dotted triangular shape to the right shows the futility boundaries and futility area of the TSA.


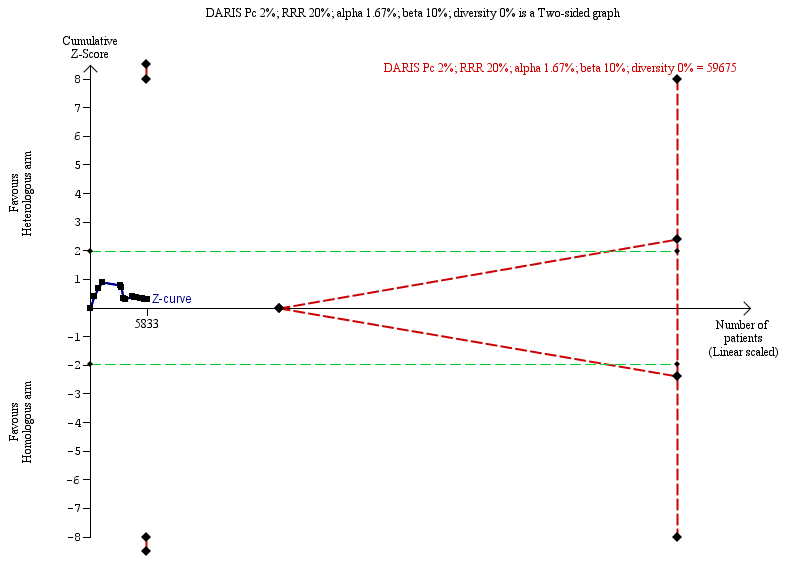


*FigS1 - Trial Sequential Analysis (TSA) for heterologous versus homologous COVID-19 vaccine regimens on all-cause mortality in healthy and immunocompromised participants.*

**TSA for heterologous versus homologous COVID-19 vaccine regimens on laboratory-confirmed symptomatic COVID-19 in healthy and immunocompromised participants**

The TSA showed that the diversity-adjusted required information size (DARIS) is 30,890 participants, calculated based on the proportion of events of 3.37 % in the homologous group; a RRR of 20%; an alpha (type Ⅰ error) of 1.67%; a beta (type Ⅱ error) of 10%; and a diversity of 0%. The cumulative z-curve does not breach the futility boundary after inclusion of 14 trials (5,677 participants), showing that we do not have a sufficient accrued number of participants to assess the effect on laboratory-confirmed symptomatic COVID-19. The TSA-adjusted CI is 0.31 to 2.98. The horizontal green dotted lines show the threshold for significance in conventional meta-analysis, at 1.96 and ̶ 1.96 of the Z value, corresponding to the P-value of 0.05. The red dotted lines above and below show the trial sequential boundaries for benefit and harm, representing the threshold for statistical significance in the TSA. The red dotted triangular shape to the right shows the futility boundaries and futility area of the TSA.


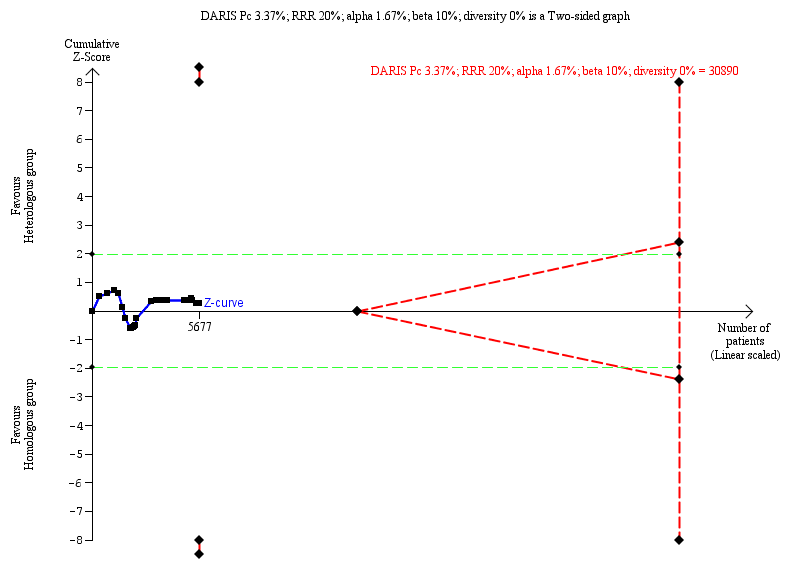


*FigS2 - TSA for heterologous versus homologous COVID-19 vaccine regimens for laboratory-confirmed symptomatic COVID-19 in healthy and immunocompromised participants.*

**TSA for heterologous versus homologous COVID-19 vaccine regimens for laboratory-confirmed severe COVID-19 in healthy and immunocompromised participants**

The TSA showed that the diversity-adjusted required information size (DARIS) is 1,214,280 participants, calculated based on the proportion of events of 0.11% in the homologous group; a RRR of 20%; an alpha (type Ⅰ error) of 1.67%; a beta (type Ⅱ error) of 10%; and a diversity of 0%. The cumulative Z-curve did not cross the conventional boundaries after inclusion of 10 trials (4,494 participants). The alpha spending function is ignored because we accrued participants less than 5% of DARIS hence no conclusive evidence was found. The horizontal green dotted lines show the threshold for significance in conventional meta-analysis, at 1.96 and ̶ 1.96 of the Z value, corresponding to the P-value of 0.05. The red lines at the right top and bottom corners, show the trial sequential boundaries for benefit or harm respectively, representing the conventional and naïve threshold for statistical significance in the TSA.


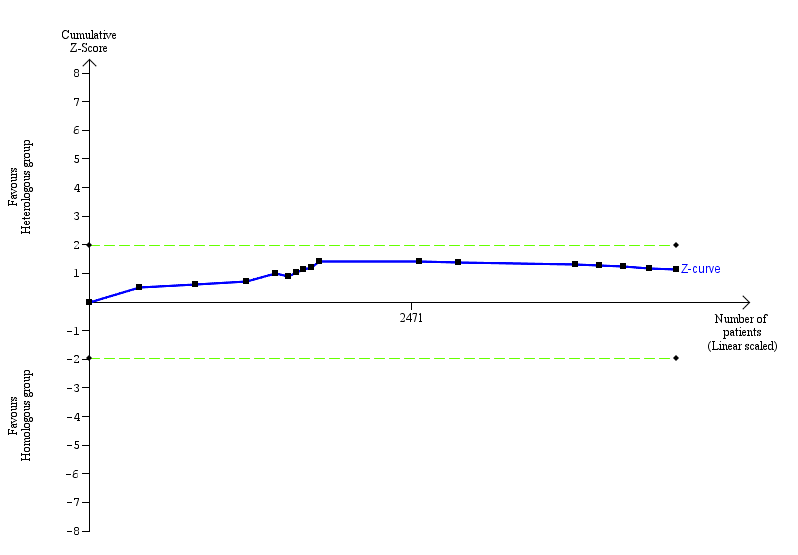


*FigS3 - TSA for heterologous versus homologous COVID-19 vaccine regimens for laboratory-confirmed severe COVID-19 in healthy and immunocompromised participants.*

**TSA for heterologous versus homologous COVID-19 vaccine regimens on serious adverse events in healthy and immunocompromised participants**

The TSA showed that the diversity-adjusted required information size (DARIS) is 167,088 participants, calculated based on the proportion of events of 0.68% in the homologous group; a RRR of 20%; an alpha (type Ⅰ error) of 1.67%; a beta (type Ⅱ error) of 10%; and a diversity of 0%. The cumulative z-curve did not cross the conventional boundaries after inclusion of 27 trials (11,384 participants). The TSA-adjusted CI is 0.10 to 19.7 shows that there is too little accrued information to make any conclusion. The horizontal green dotted lines show the threshold for significance in conventional meta-analysis, at 1.96 and ̶ 1.96 of the Z value, corresponding to the P-value of 0.05. The red lines at the right top and bottom corners, show the trial sequential boundaries for benefit or harm respectively, representing the threshold for statistical significance in the TSA.


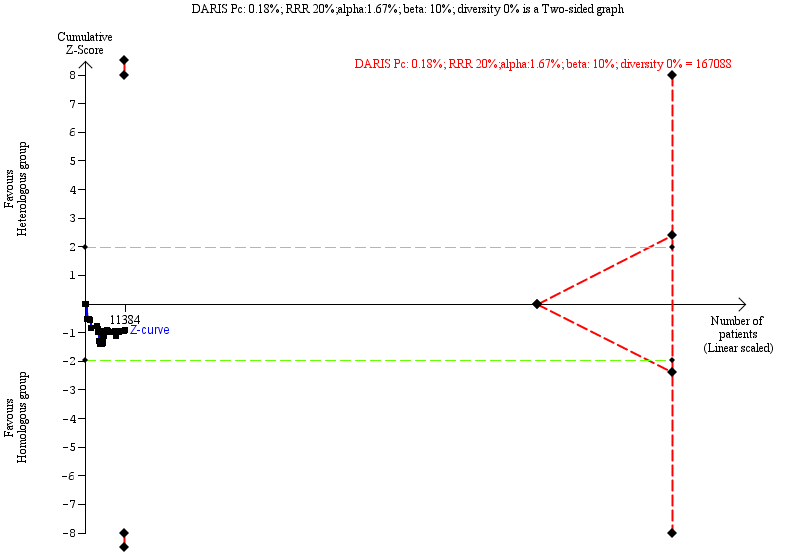


*FigS4 - TSA for heterologous versus homologous COVID-19 vaccine regimens for serious adverse events in healthy and immunocompromised participants.*

**TSA for heterologous versus homologous COVID-19 vaccine regimens for adverse events considered non-serious in healthy and immunocompromised participants**

The TSA showed that the diversity-adjusted required information size (DARIS) is 69,880 participants, calculated based on the proportion of events of 32.7% in the homologous group; a RRR of 20%; alpha (type Ⅰ error) of 1.67%; beta (type Ⅱ error) of 10%; and diversity of 96.4%. The cumulative Z-curve did not cross the boundary for harm nor the boundary of benefit of heterologous vaccines after inclusion of 20 trials (10,008 participants). The TSA-adjusted CI is 0.73 to 2.16 showing that there is too little accrued information to make any conclusion. The horizontal green dotted lines show the threshold for significance in conventional meta-analysis, at 1.96 and ̶ 1.96 of the Z value, corresponding to the P-value of 0.05. The red dotted lines from the left top and bottom corners, show the trial sequential boundaries for benefit or harm, respectively, representing the threshold for statistical significance in the TSA. The red dotted triangular shape to the right shows the futility boundaries and futility area of the TSA.


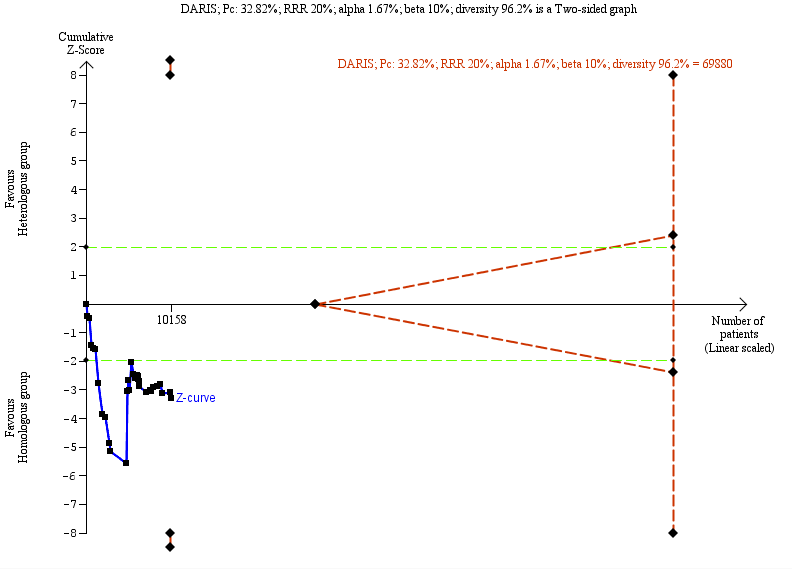


*FigS5 - TSA for heterologous versus homologous COVID-19 vaccine regimens for adverse events considered non-serious in healthy and immunocompromised participants.*

## Risk of bias assessment

Safety outcomes


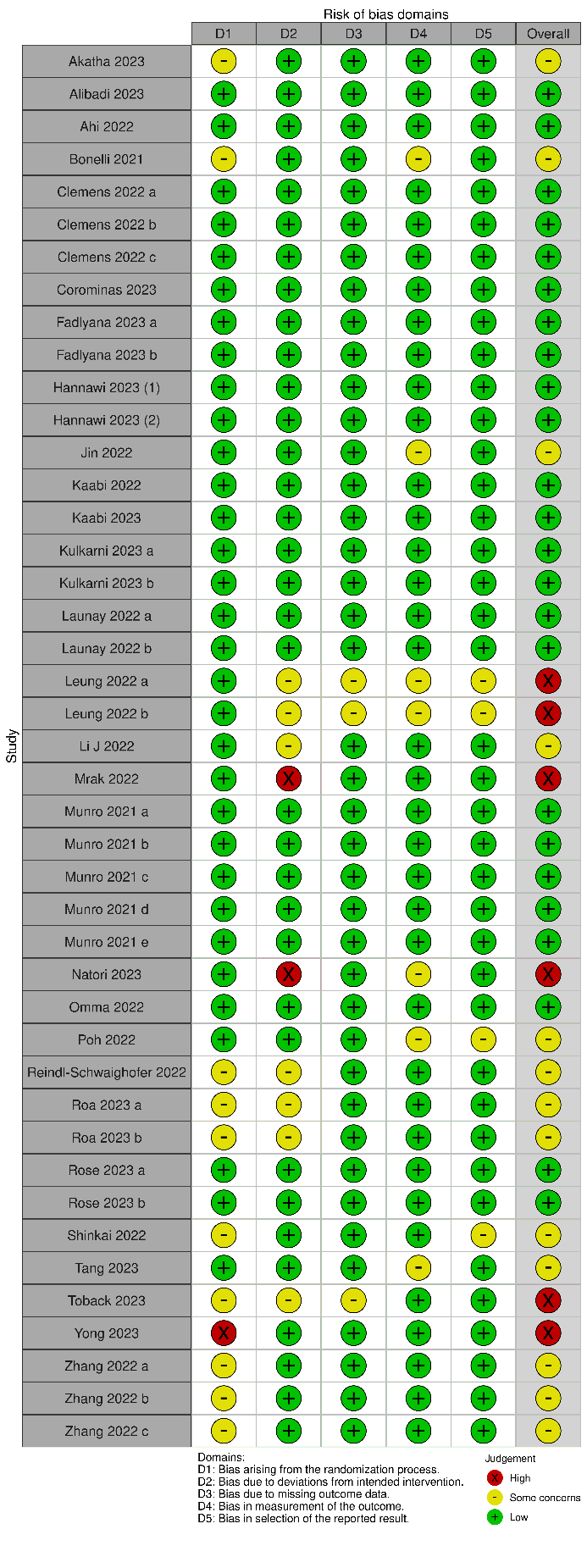


*FigS6 - Risk of bias assessment for safety outcomes.*

**Summary plot: safety outcomes**


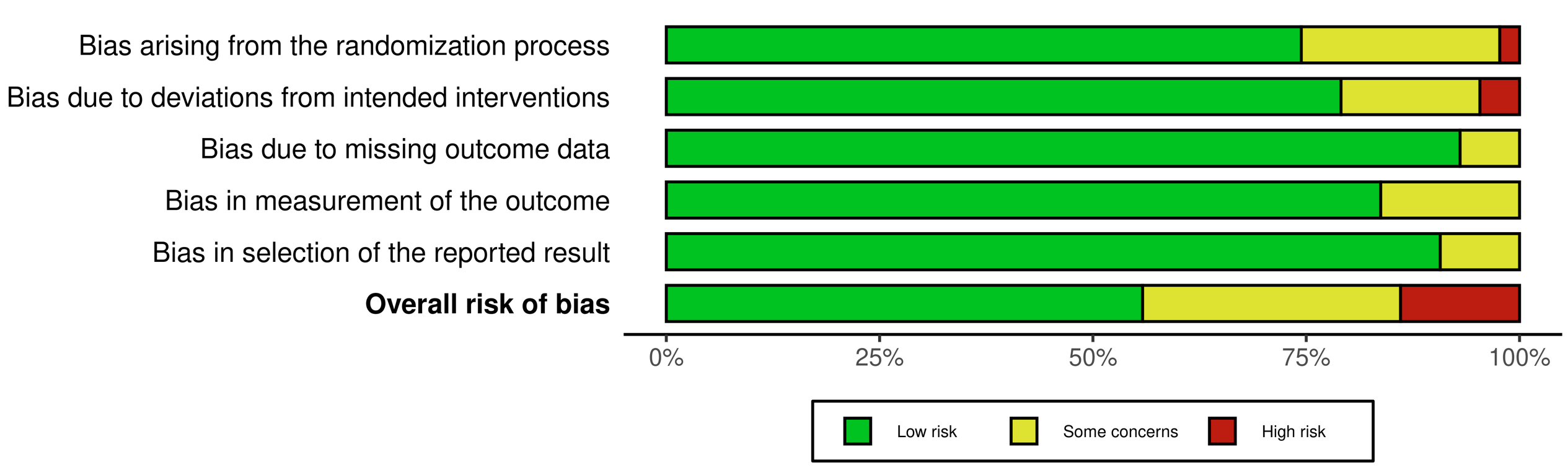


*FigS7 - Summary plot for safety outcomes.*

**All-cause mortality**


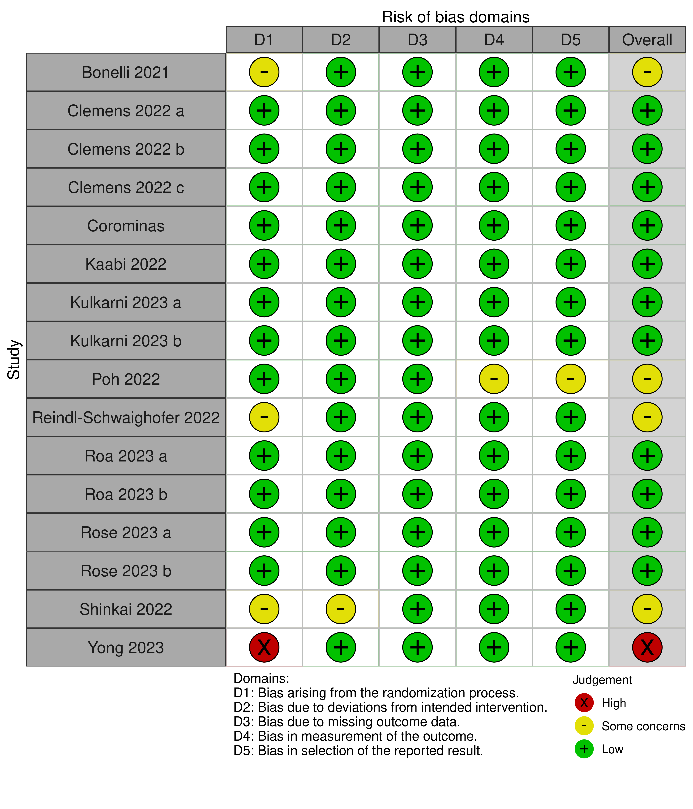


*FigS8 - Risk of bias for all-cause mortality.*

**Summary plot: all-cause mortality**


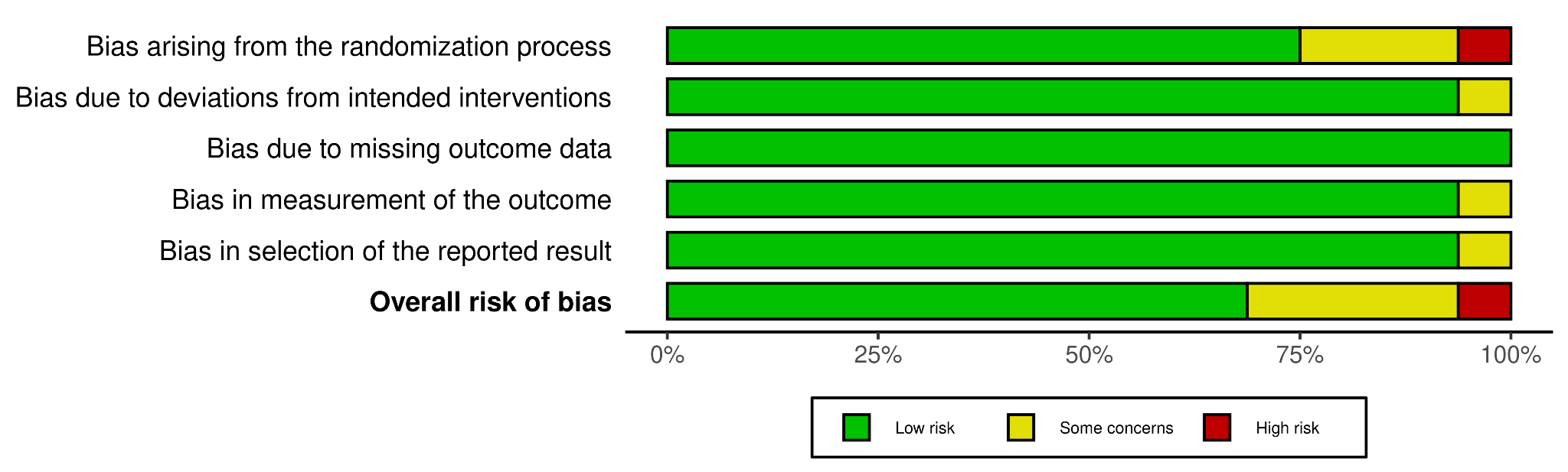


*FigS9 - Summary plot for all-cause mortality.*

**Vaccine Efficacy**


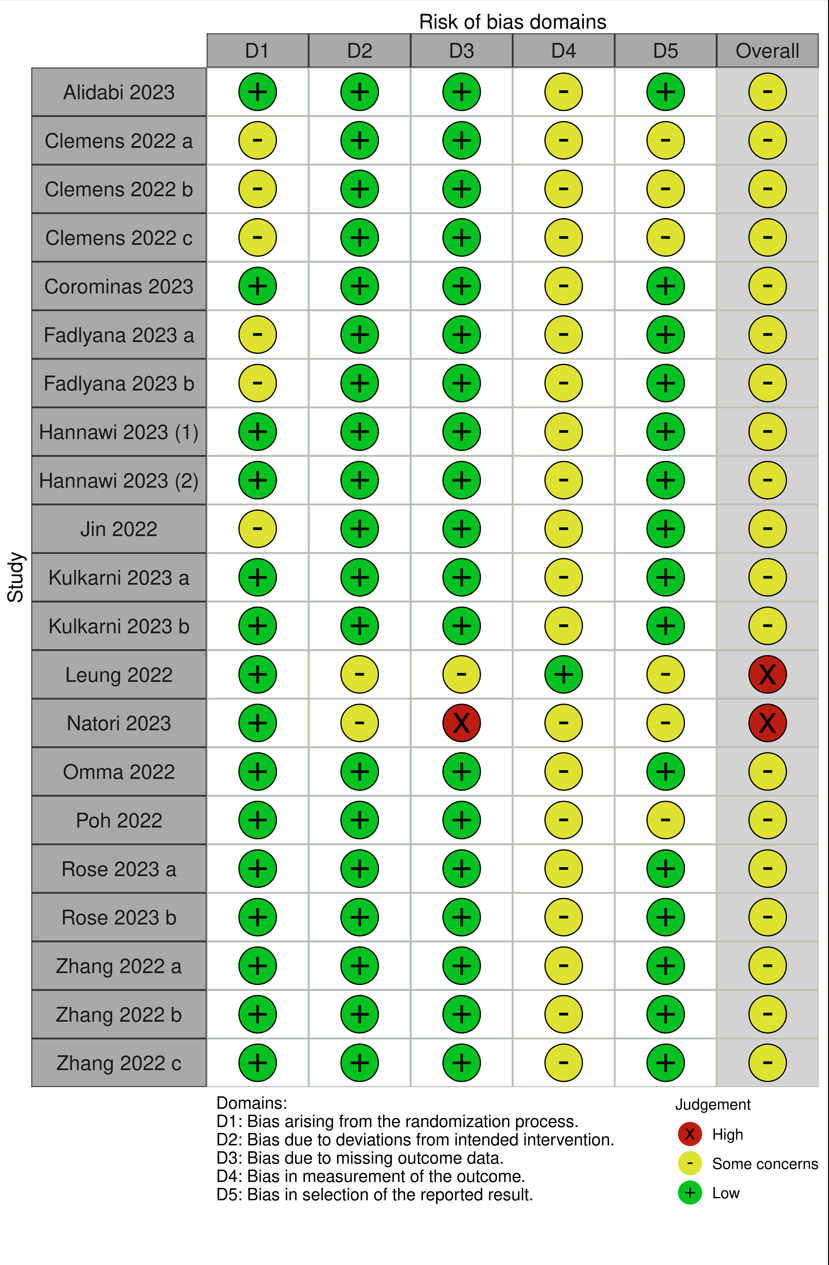


*FigS10 - Risk of bias assessment for vaccine Efficacy.*

**Summary plot for vaccine efficacy**


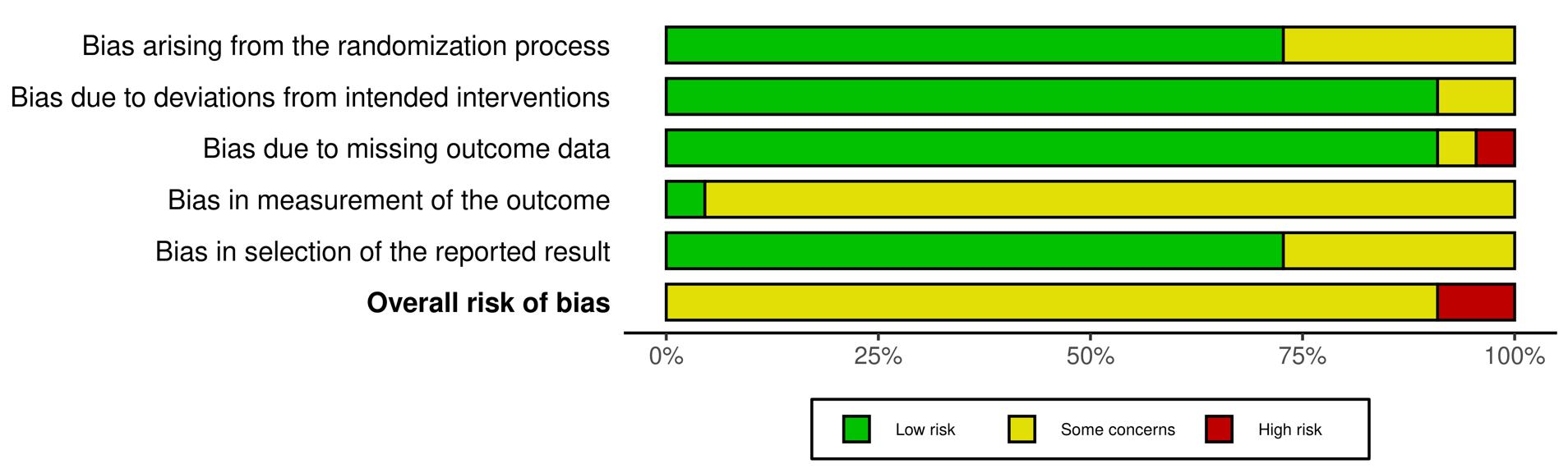


## *FigS11 - Summary plot Vaccine efficacy.*

## Additional supplementary figures

**Subgroup analysis for across different heterologous booster vaccine platforms**

**All-cause mortality**


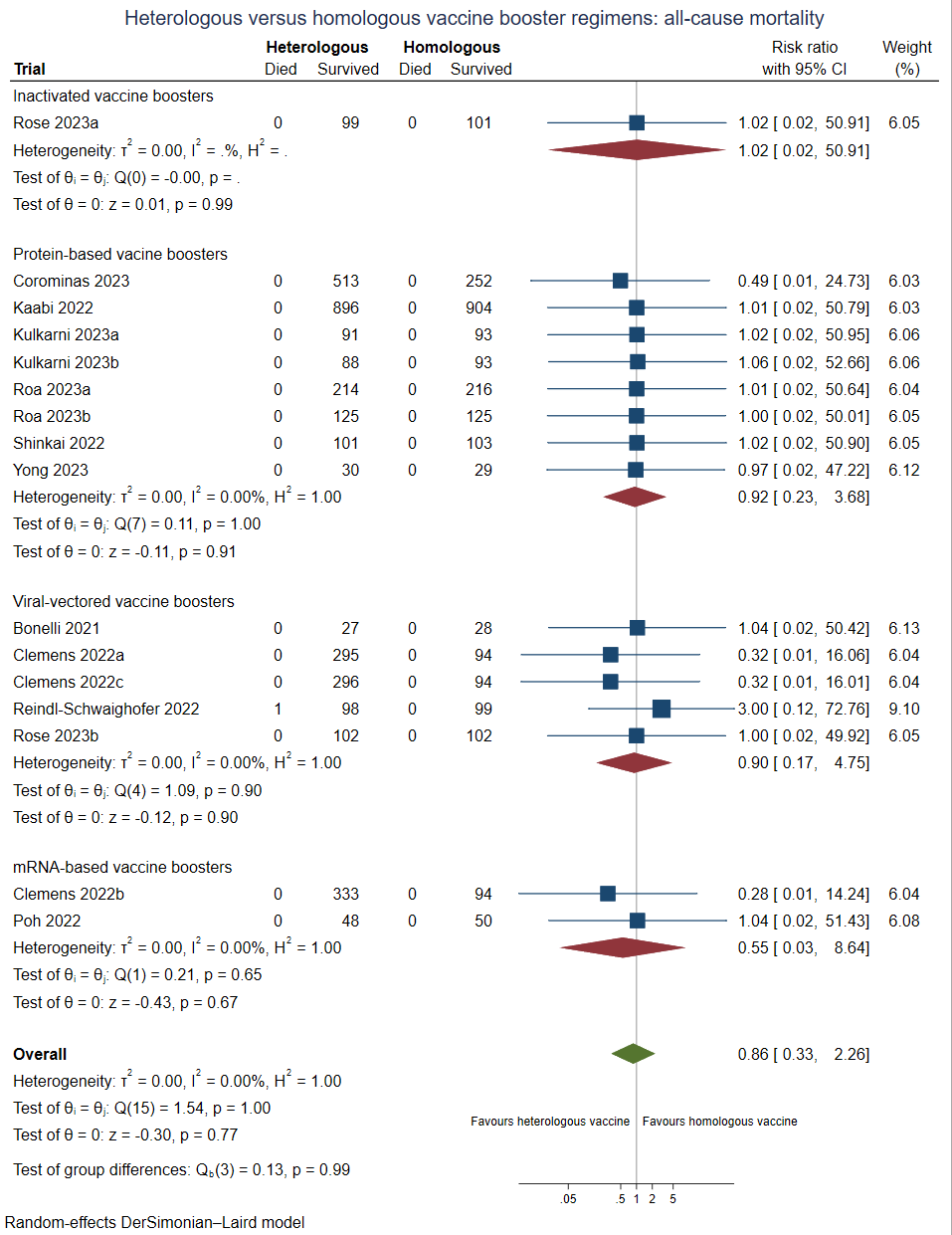


*Figure S12 Subgroup analysis for across different heterologous booster vaccine platforms: all-cause mortality.*

**Laboratory confirmed symptomatic COVID-19**

**
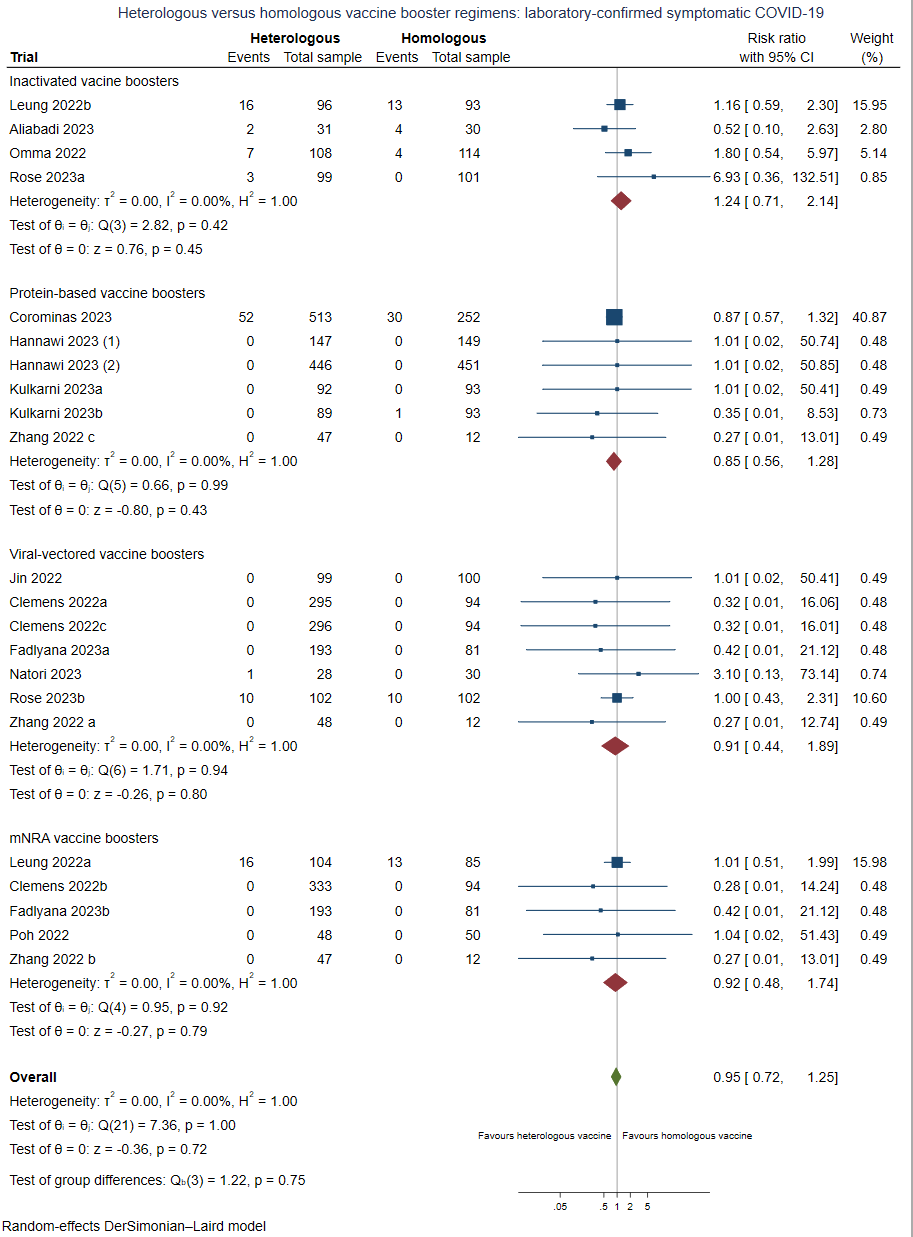
**

*Figure S13 Subgroup analysis for across different heterologous booster vaccine platforms: laboratory-confirmed symptomatic COVID-19.*

**Laboratory confirmed severe COVID-19**


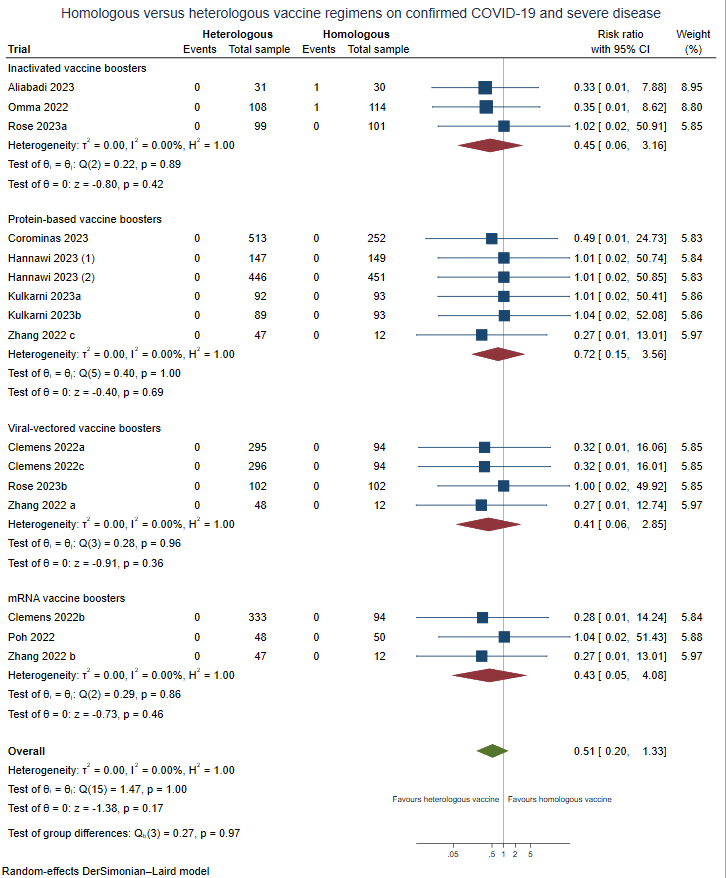


*Figure S14 Subgroup analysis for across different heterologous booster vaccine platforms: laboratory-confirmed severe COVID-19.*

**Serious adverse events**

**
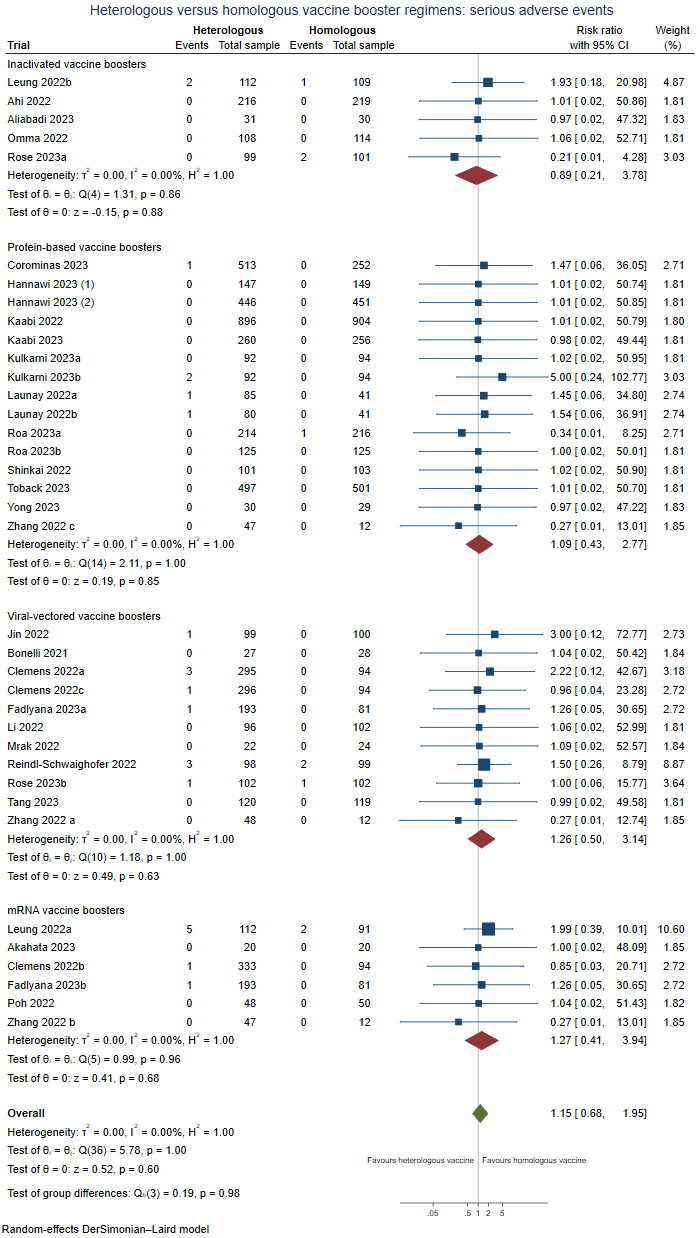
**

*Figure S15 Subgroup analysis for across different heterologous booster vaccine platforms: Serious adverse events.*

**Adverse event considered non-serious**

**
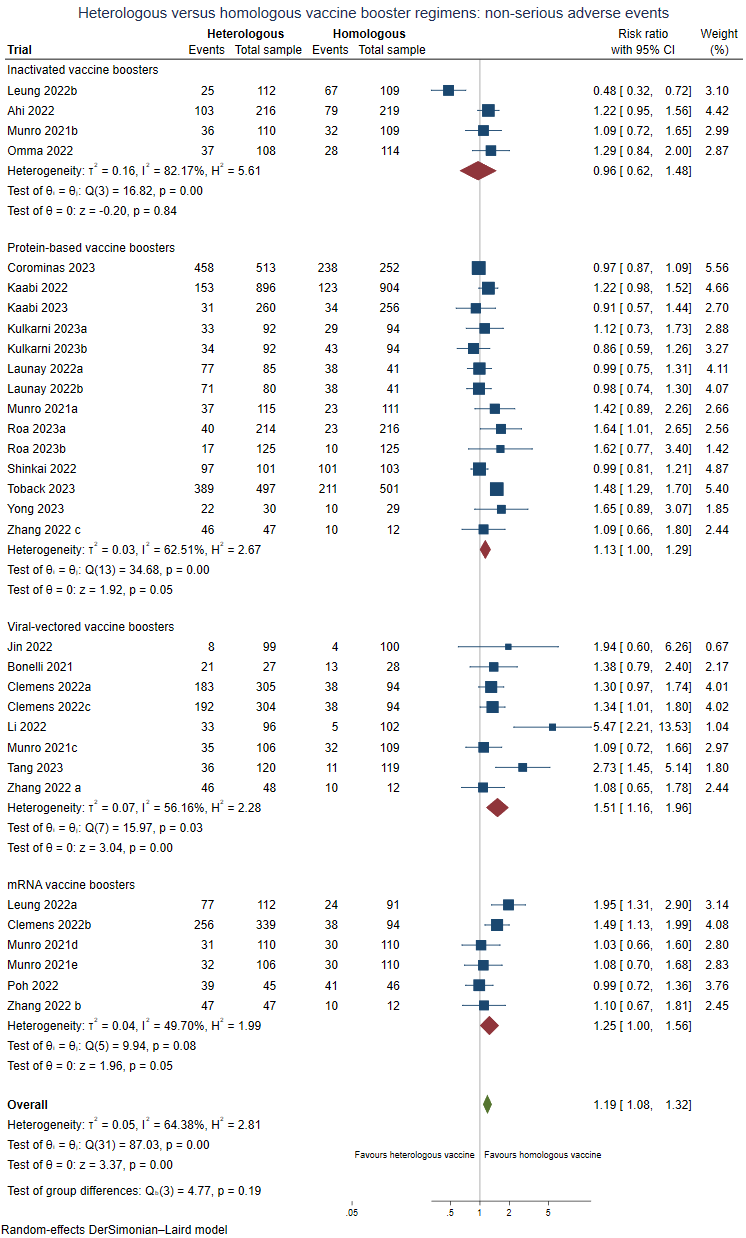
**

*Figure S16 Subgroup analysis for across different heterologous booster vaccine platforms: Adverse events considered non serious.*

**All-cause mortality**

**
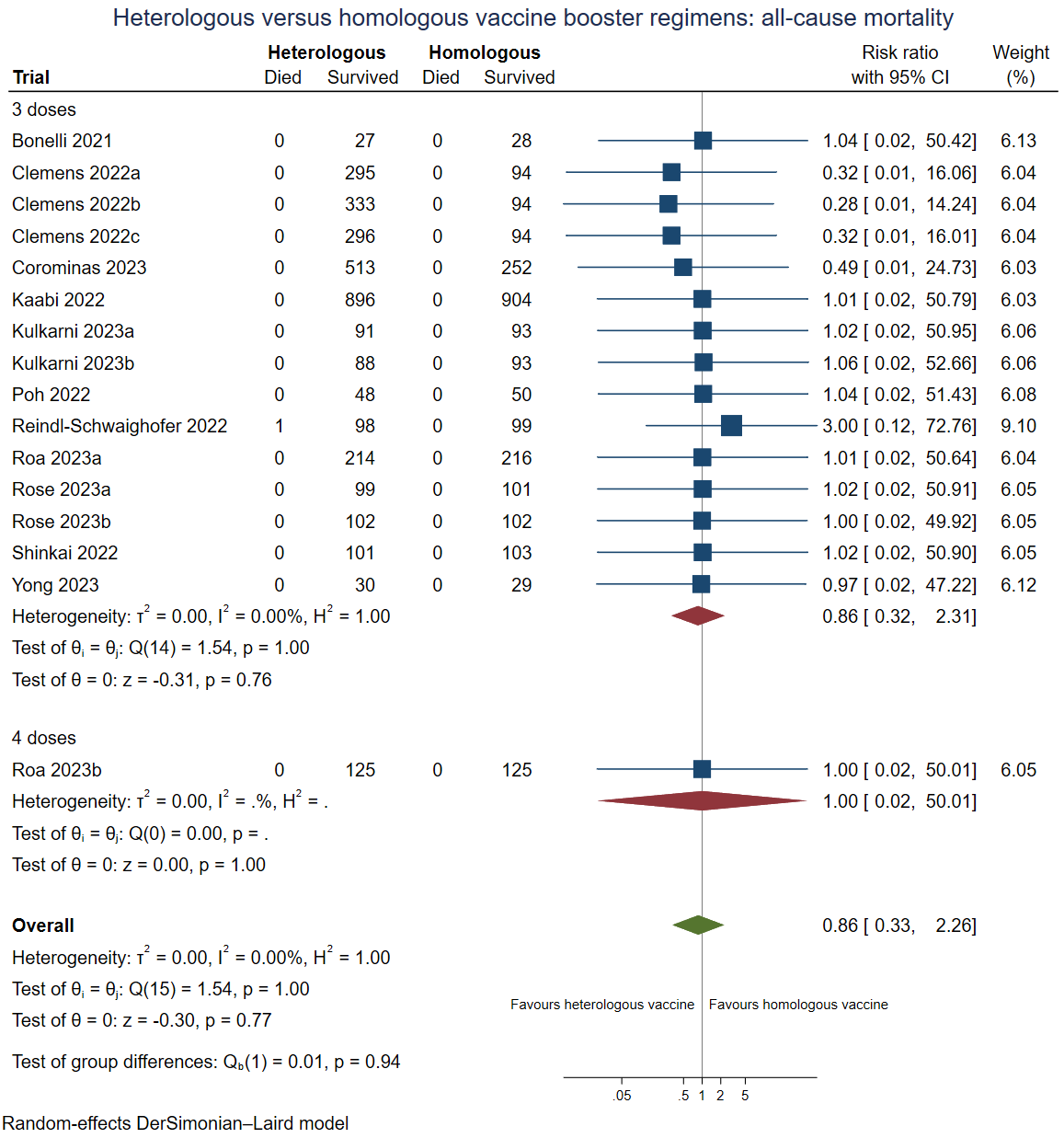
**

*Figure S17 Subgroup analysis across different doses: All-cause mortality*

**Serious adverse events**

**
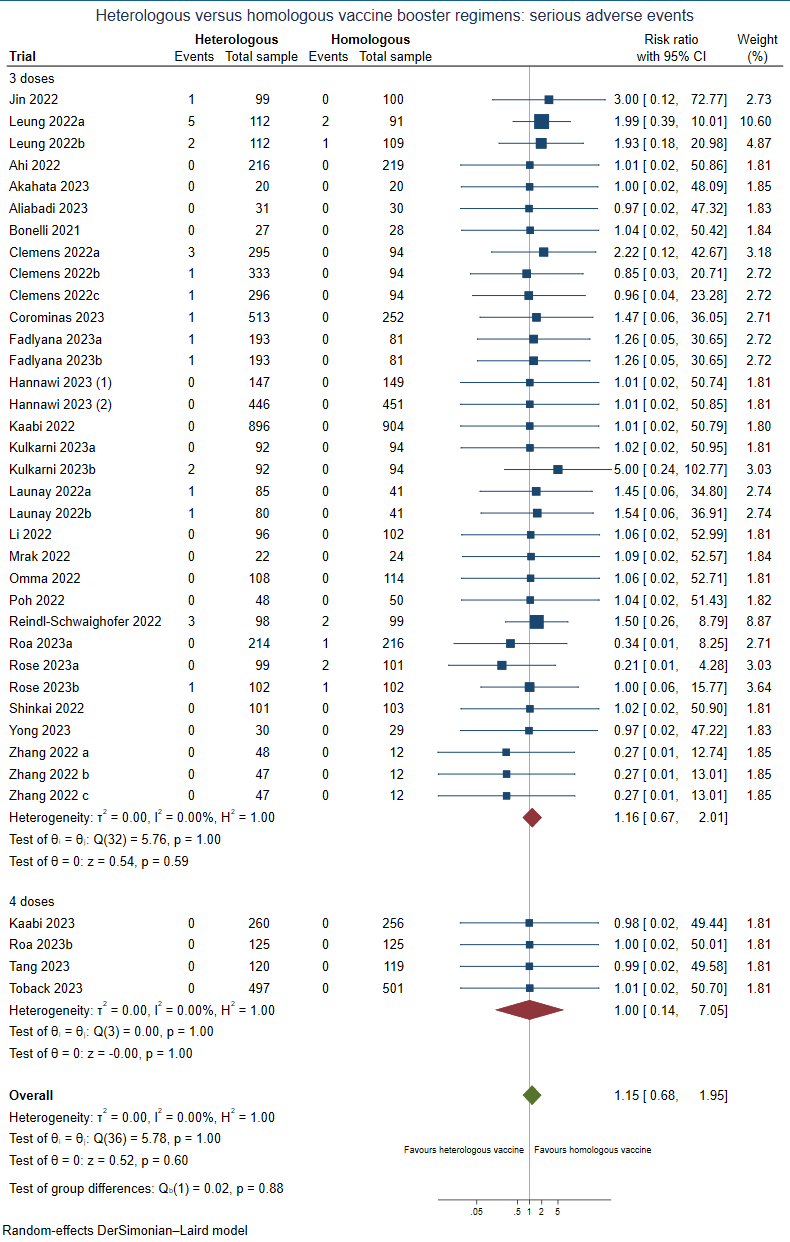
**

*Figure S18 Subgroup analysis across different doses: Serious adverse events.*

**Adverse events considered non-serious**

**
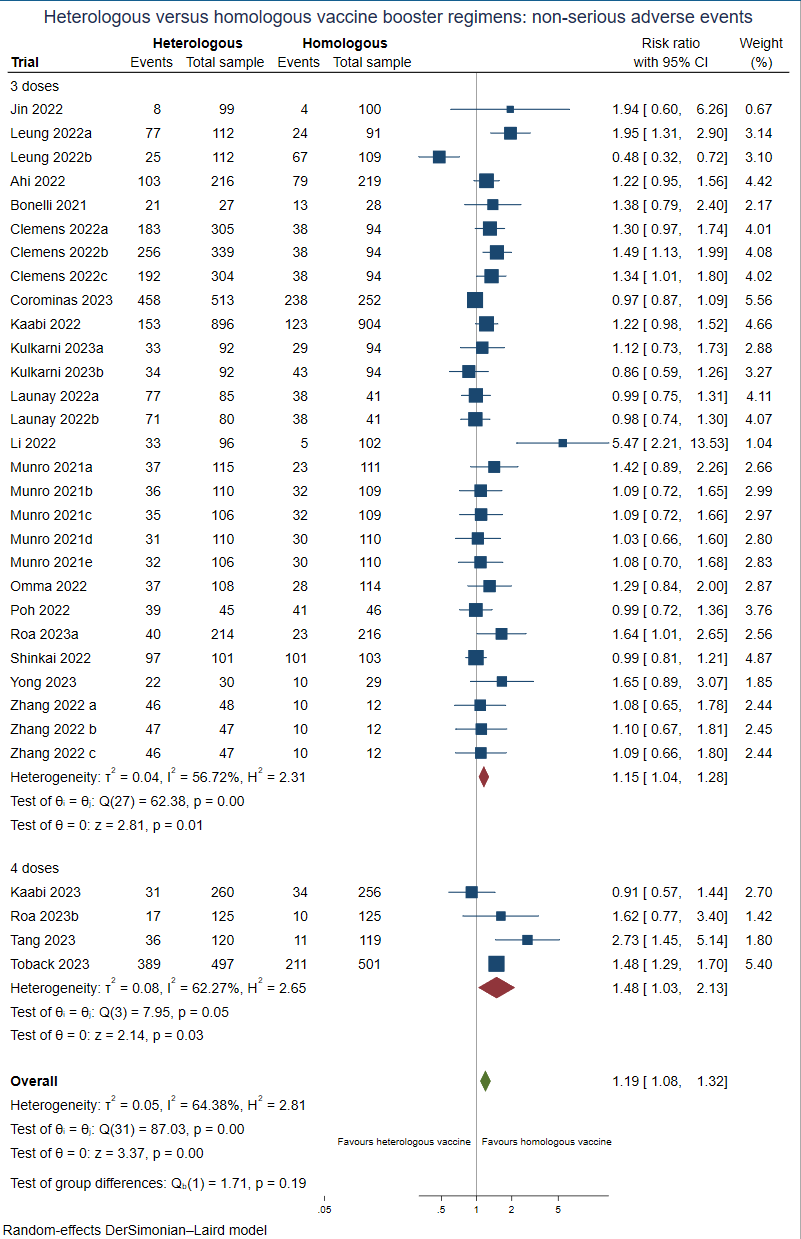
**

*Figure S19 Subgroup analysis across different doses: Adverse events considered non-serious.*

**Sub-group analysis by follow-up**

**
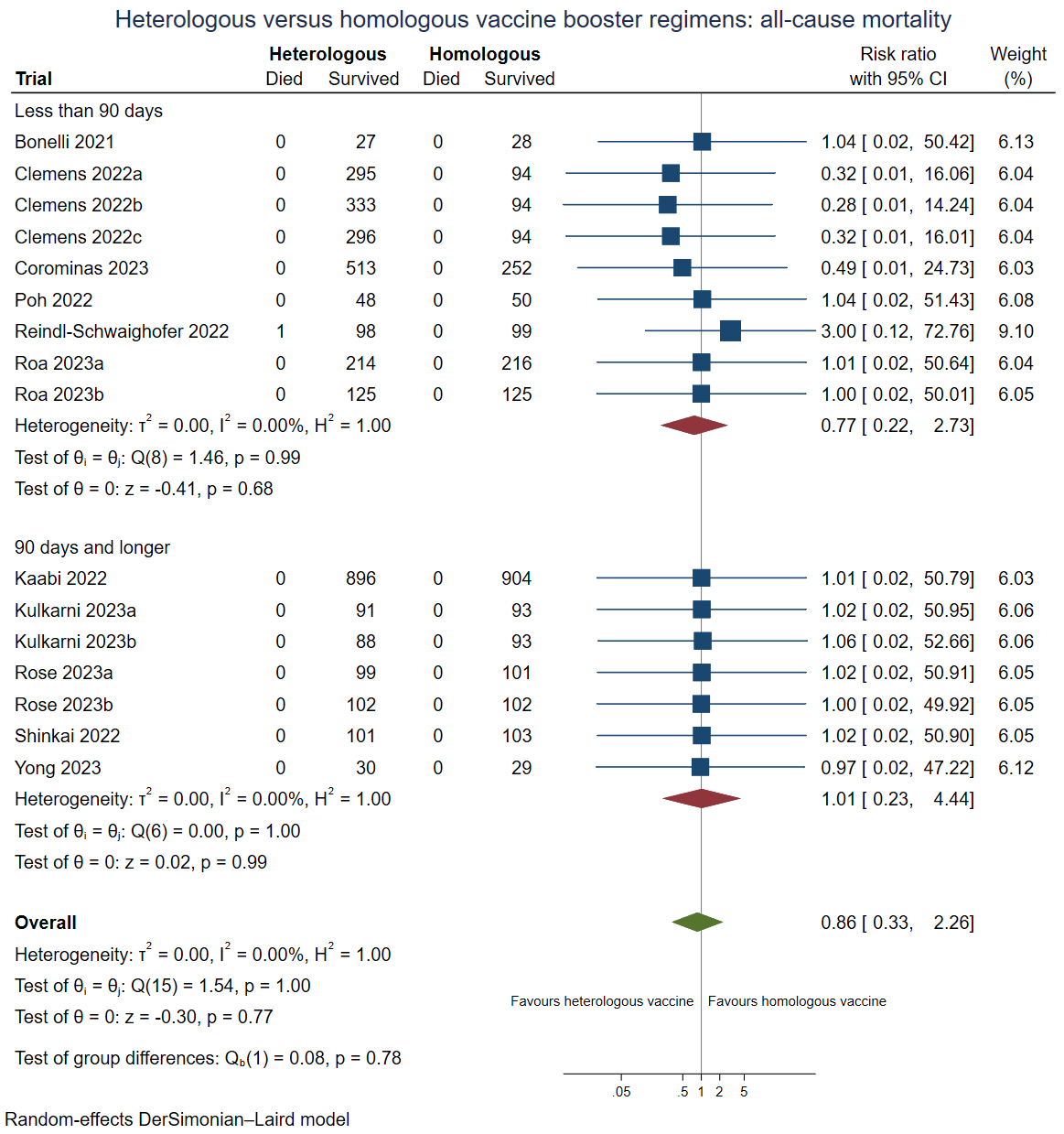
***Figure S20 Subgroup analysis across different periods of follow-up: all-cause mortality.*

**Laboratory-confirmed symptomatic COVID-19**

**
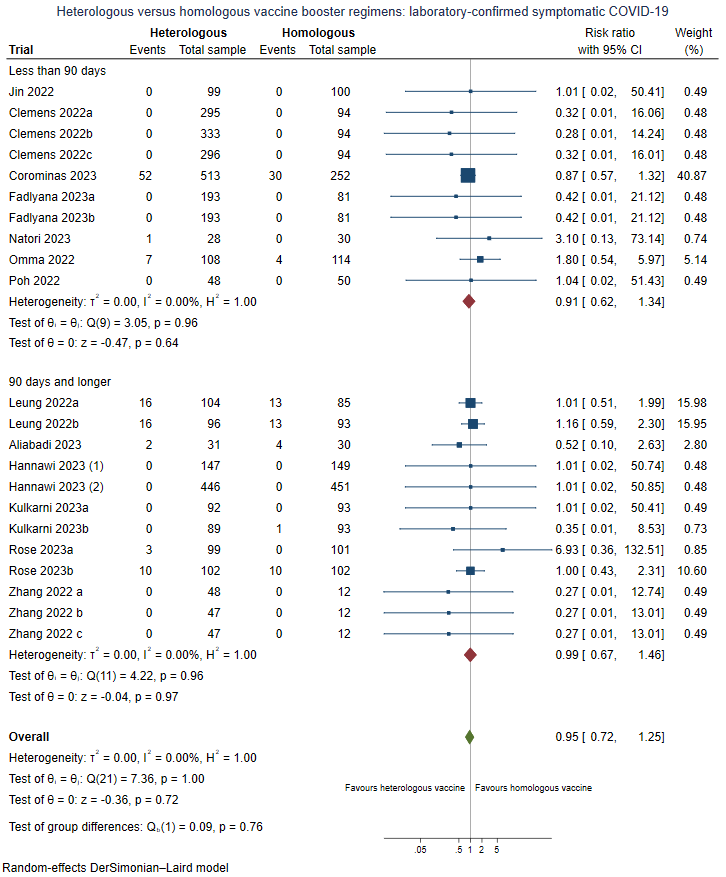
**

*Figure S21 Subgroup analysis across different periods of follow-up: laboratory-confirmed symptomatic COVID-19.*

**Laboratory-confirmed severe COVID-19**

**
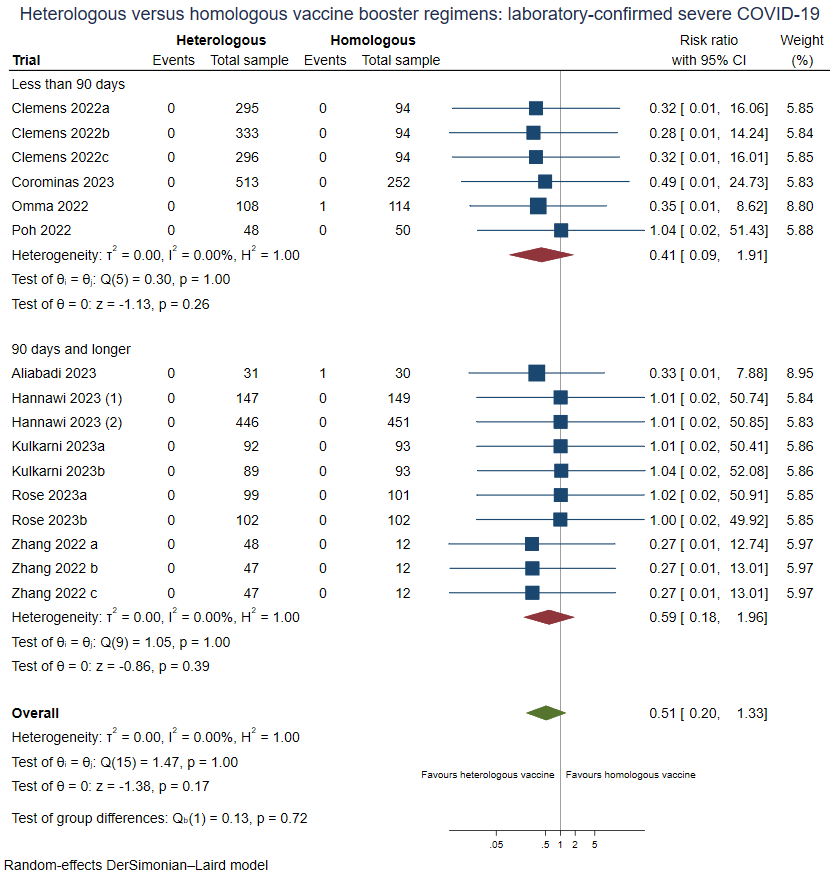
**

*Figure S22 Subgroup analysis across different periods of follow-up: laboratory-confirmed severe COVID-19.*

**Serious adverse event**

**
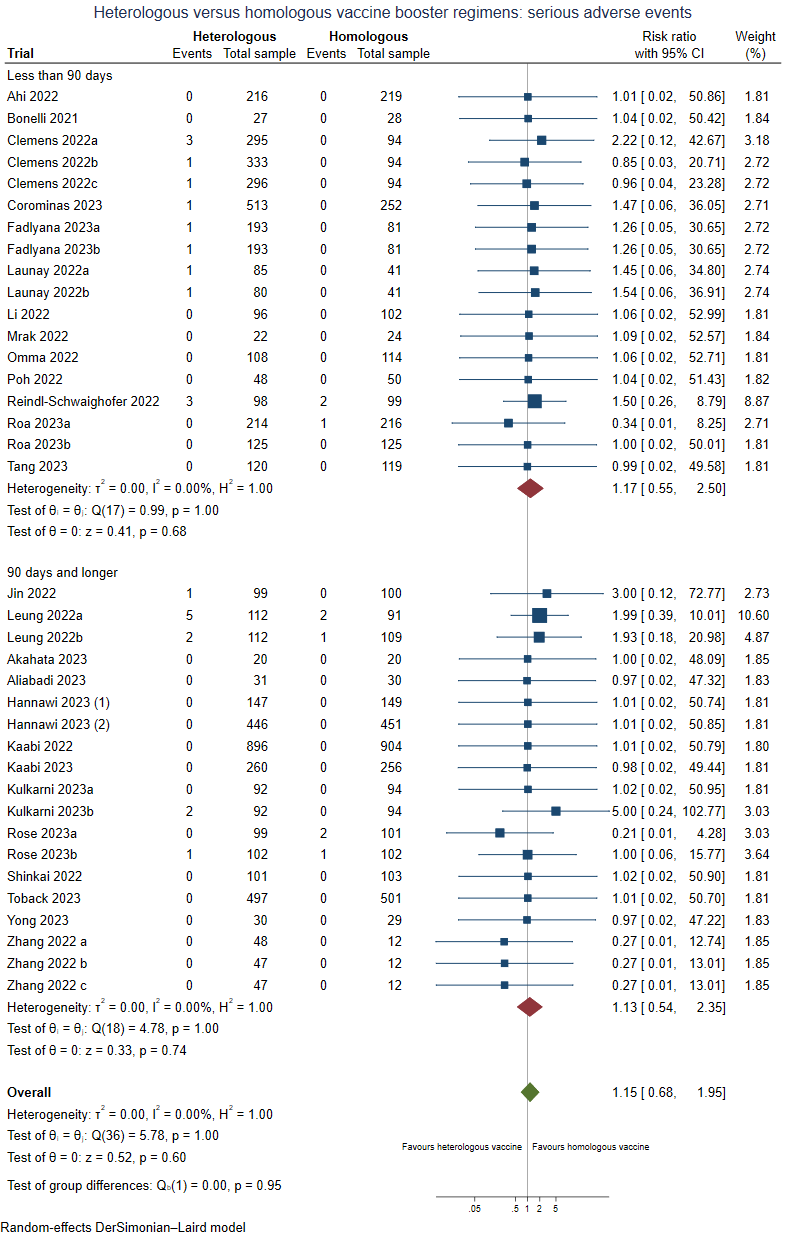
**

*Figure S23: Subgroup analysis across different periods of follow-up: serious adverse events.*

**Sub-group analysis by risk of bias**

**All-cause mortality**

**
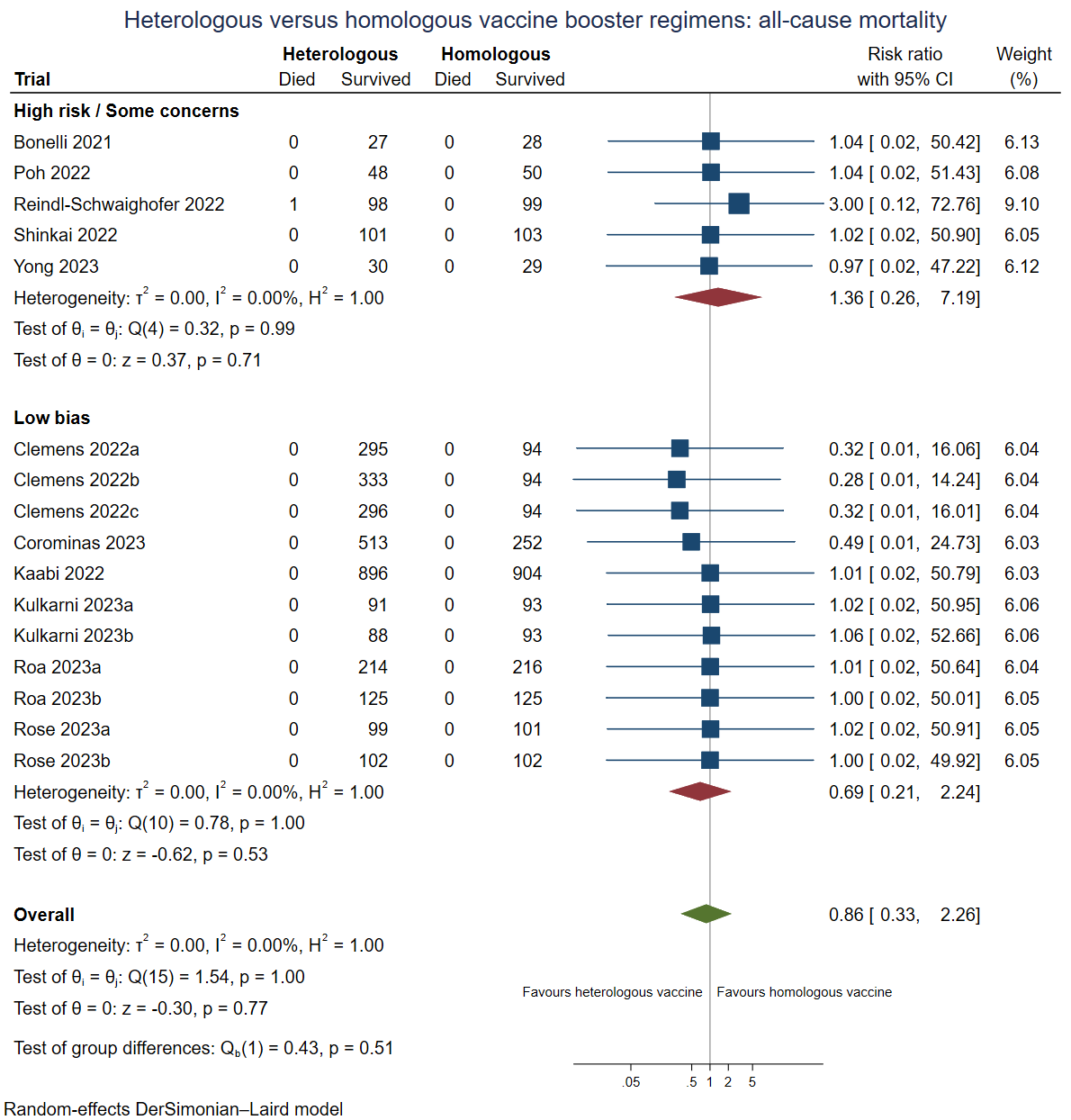
**

*Figure S24: Subgroup analysis by risk of bias: all-cause mortality.*

**Serious adverse event**


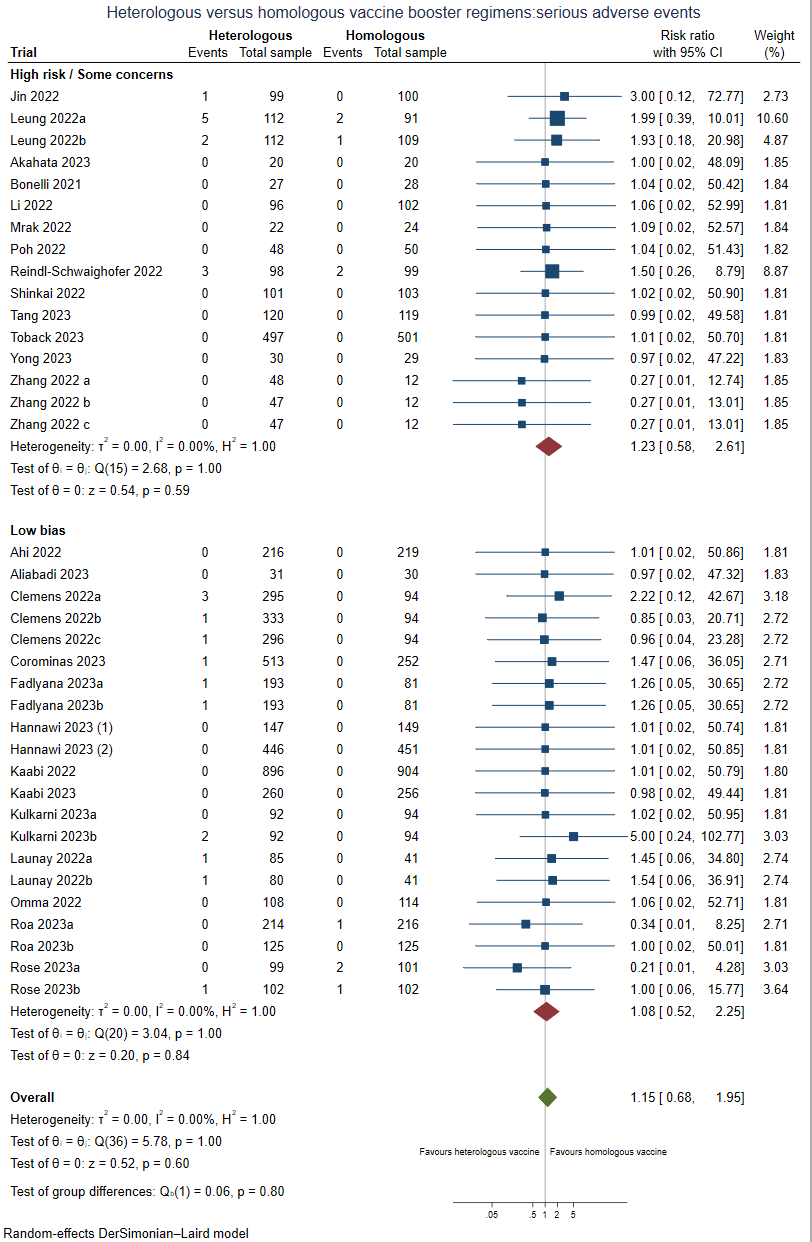


*Figure S25 Subgroup analysis by risk of bias: serious adverse events.*

**Adverse event considered non-serious**

**
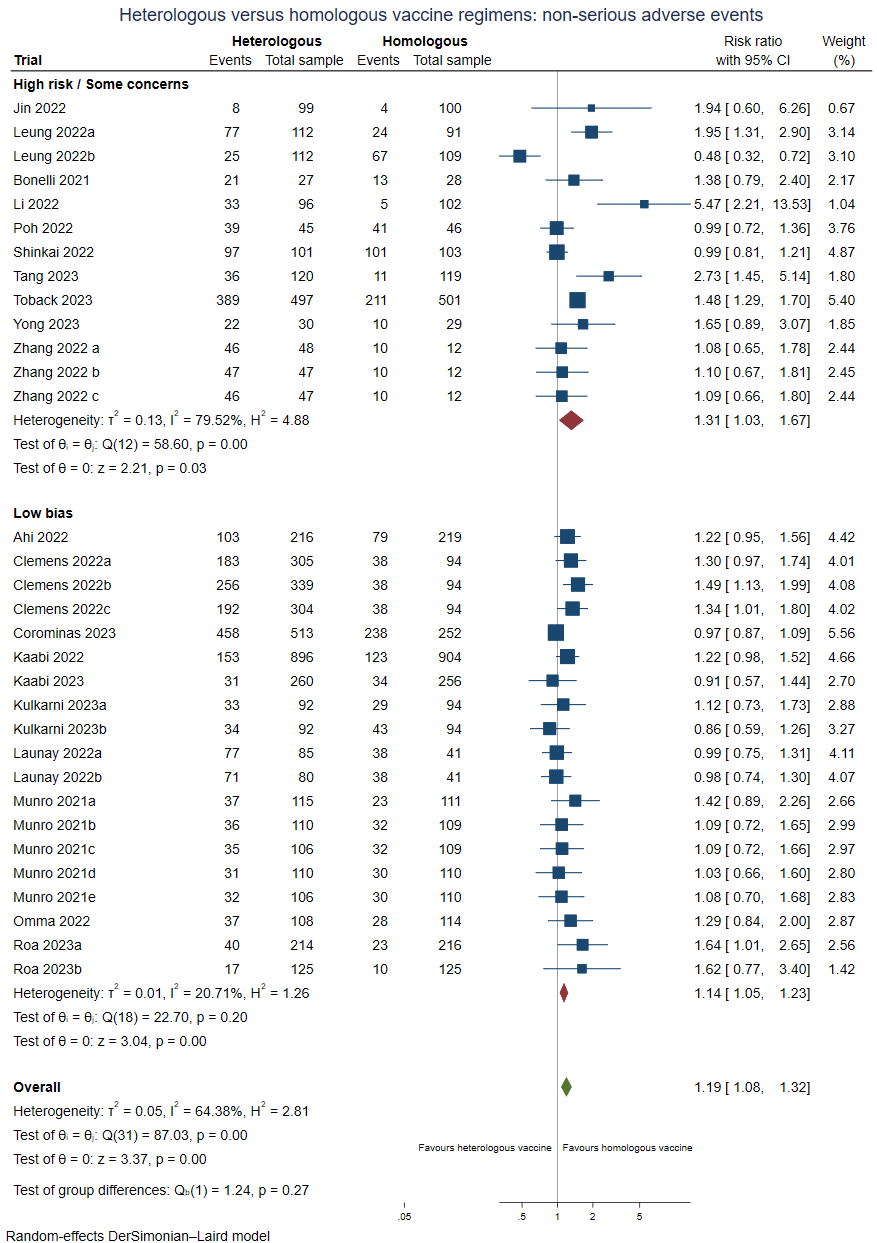
**

*Figure S26 Subgroup analysis by risk of bias: adverse events considered non-serious.*

**Sub-group analysis by health status**

**All-cause mortality**

**
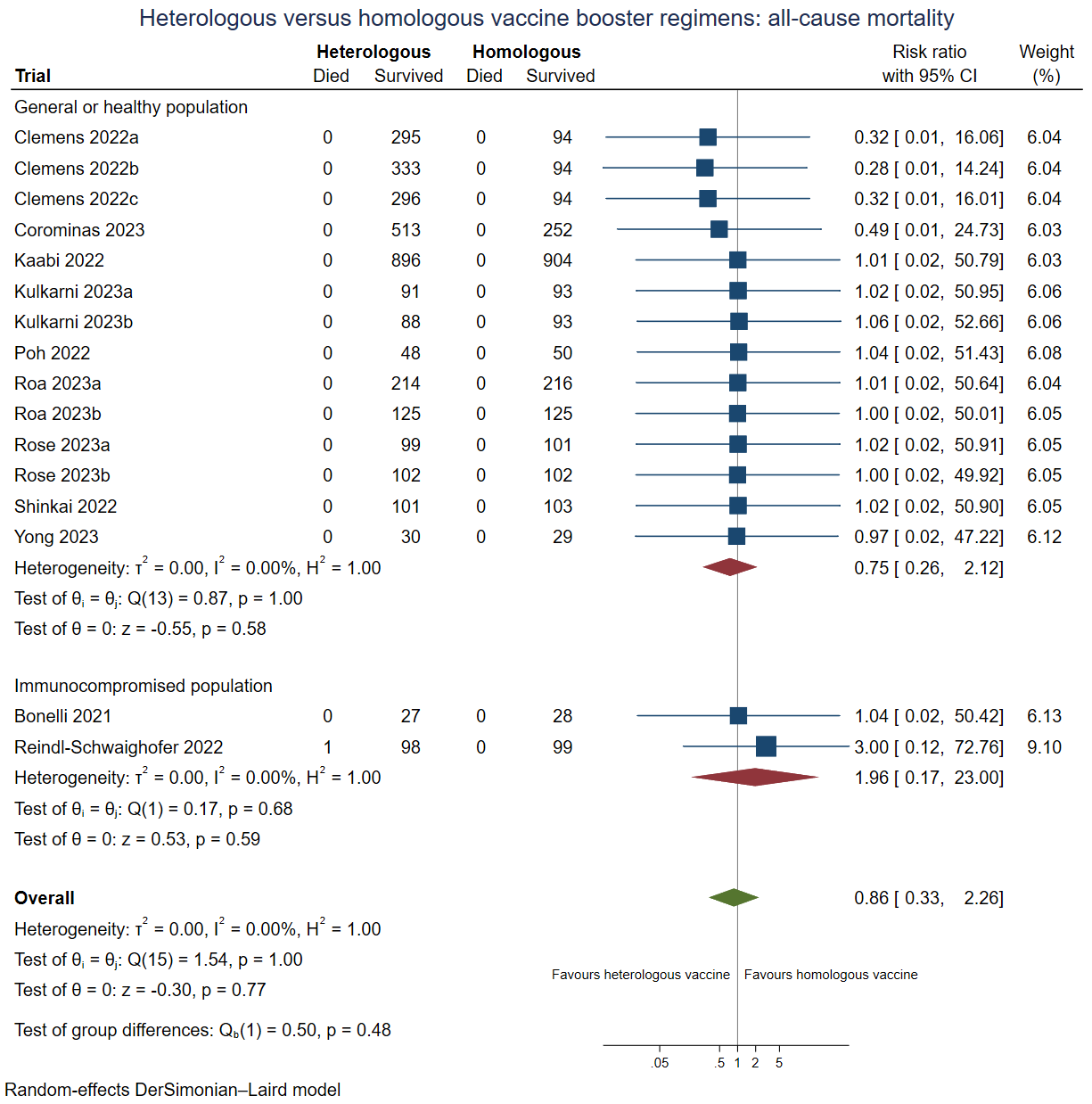
**

*Figure S27 Subanalysis by health status: All-cause mortality.*

**Laboratory-confirmed symptomatic COVID-19**

**
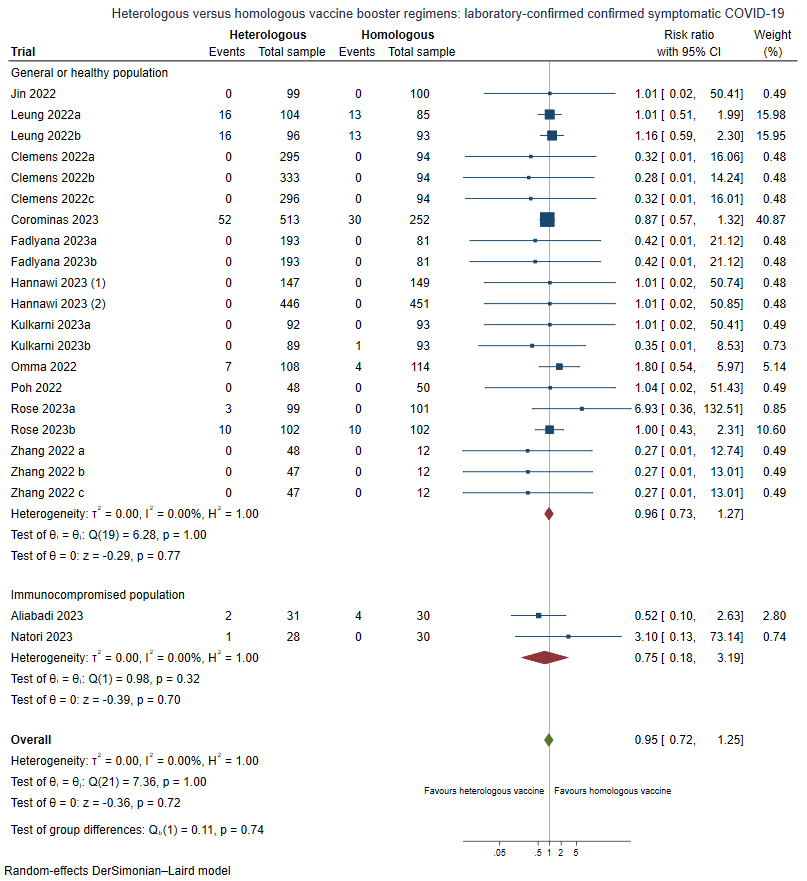
**

*Figure S28 Subanalysis by health status: laboratory-confirmed symptomatic COVID-19.*

**Serious adverse events**

**
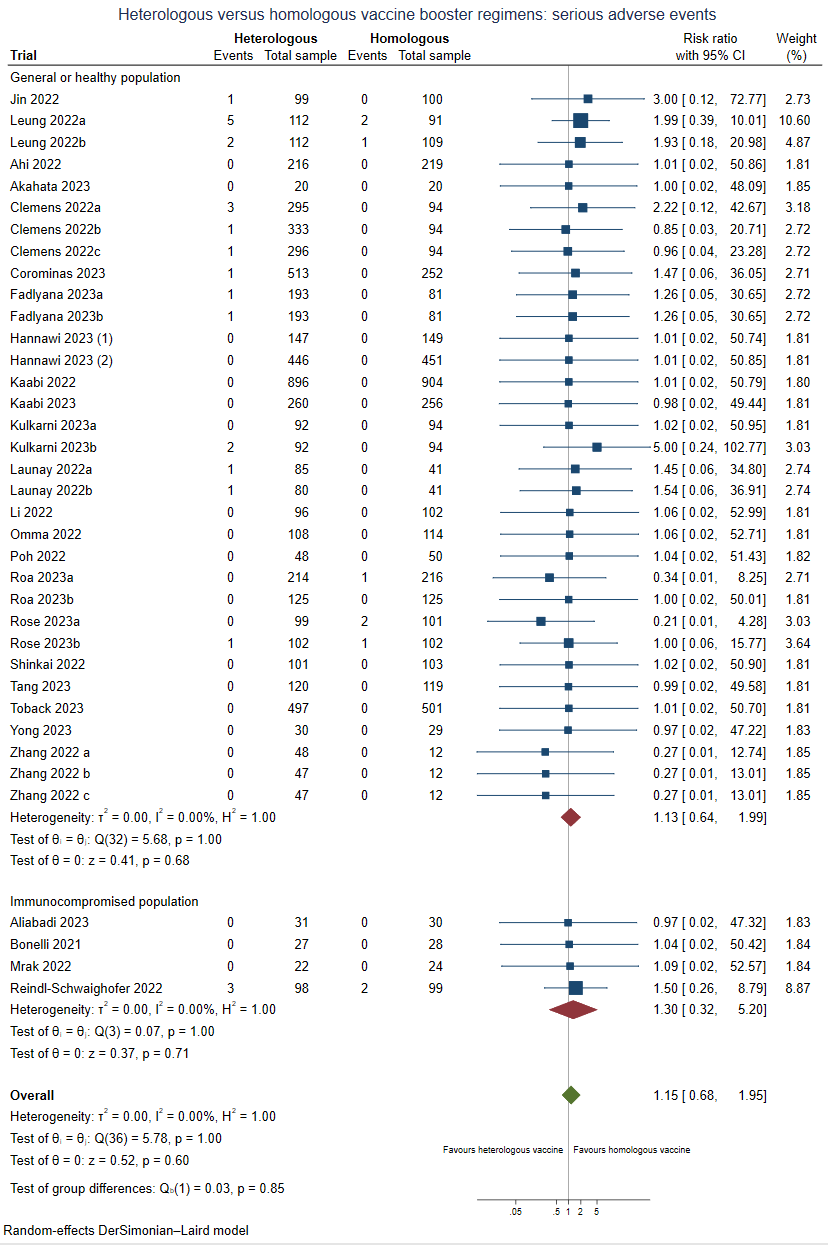
**

*Figure S29 Subanalysis by health status: serious adverse events.*

**Adverse events considered non-serious**

**
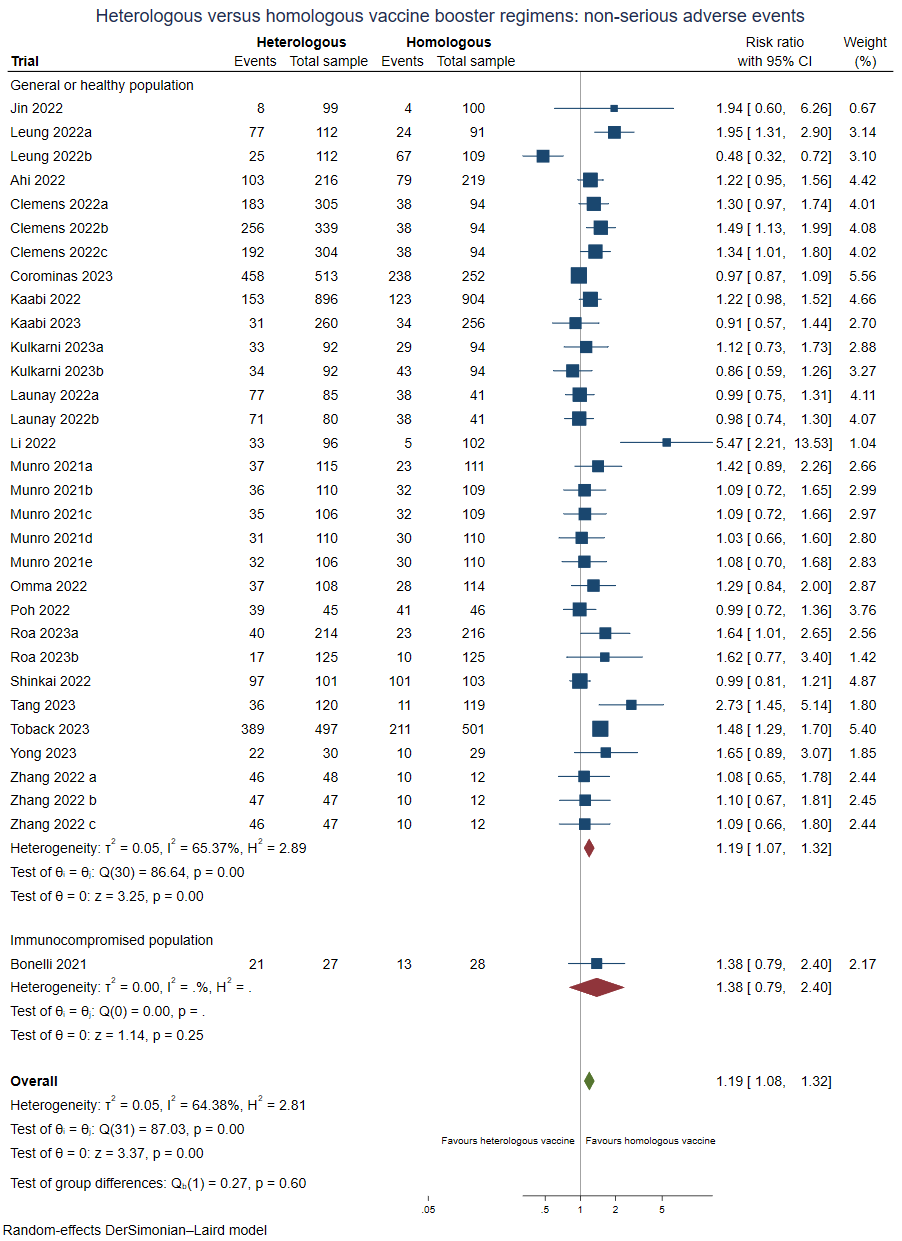
**

*Figure S30 Subanalysis by health status: adverse events non-serious.*

**All-cause mortality**

**
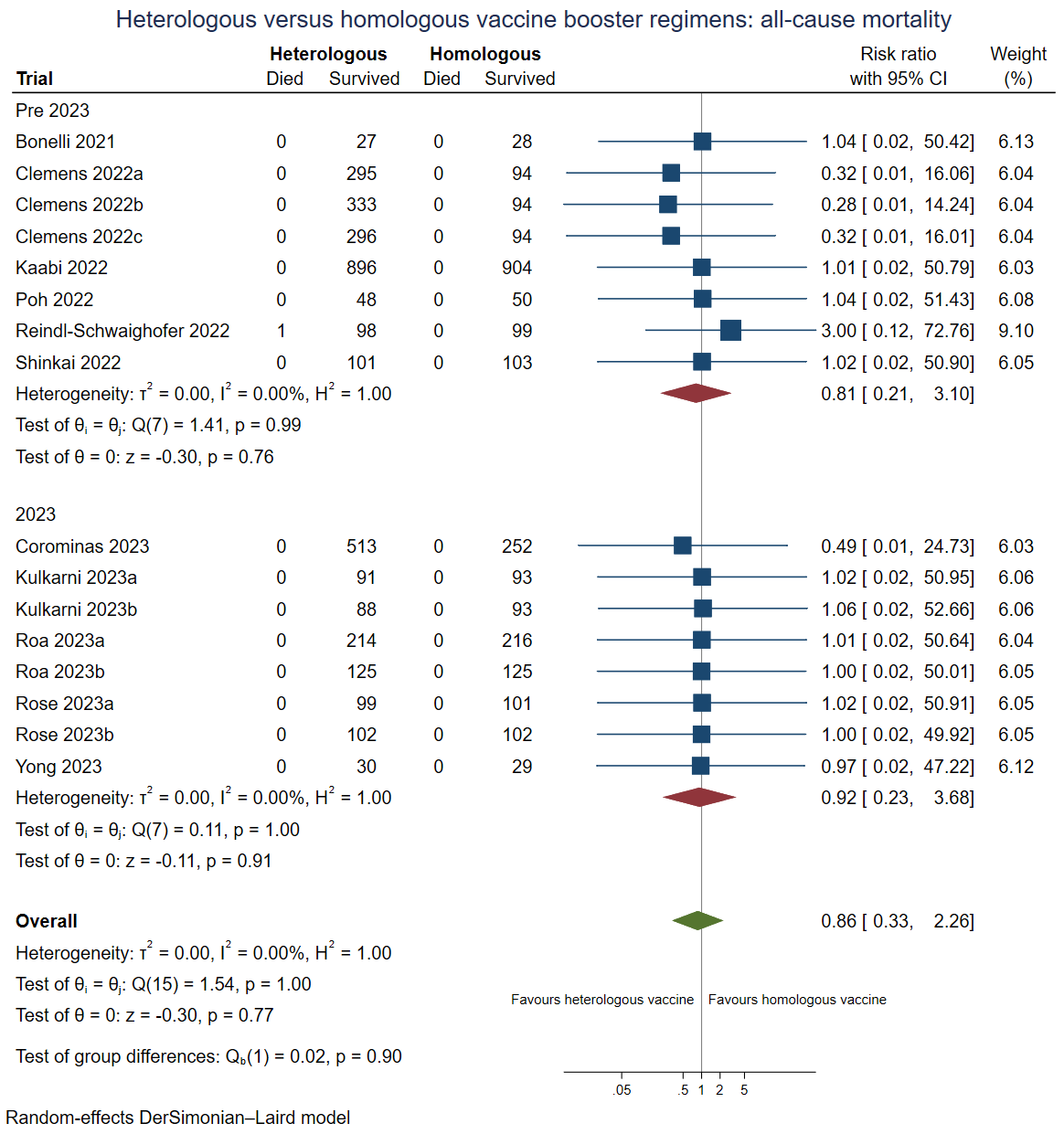
**

*Figure S31 Subanalysis for all-cause mortality by pre 2023 and in 2023 to capture the predominance of Omicron subvariant XBB.*

**Laboratory-confirmed symptomatic COVID-19**

**
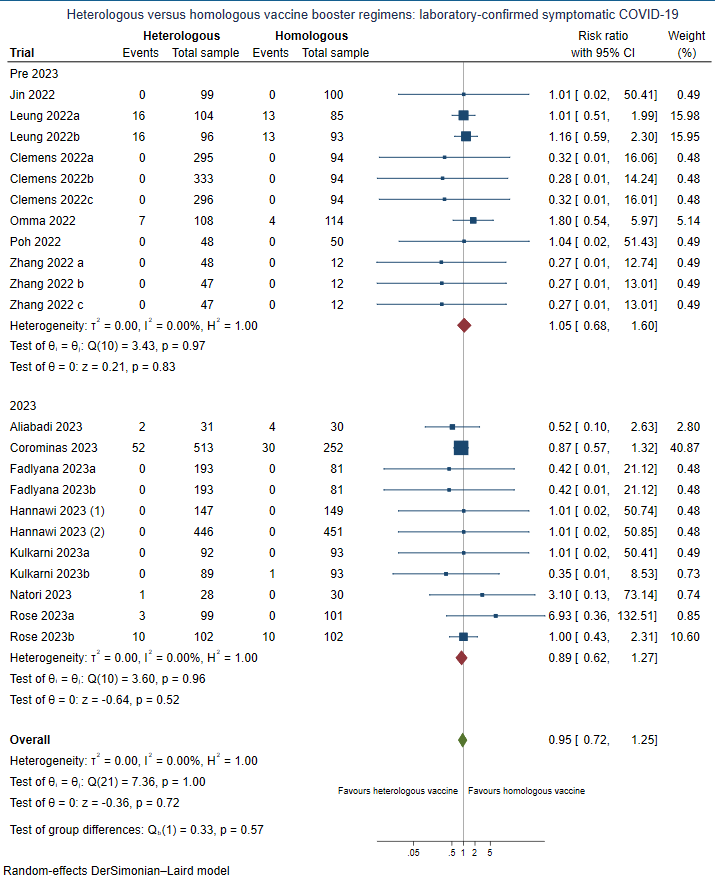
**

*Figure S32 Subanalysis for laboratory-confirmed symptomatic COVID-19 by pre 2023 and in 2023 to capture the predominance of Omicron subvariant XBB.*

**Laboratory-confirmed severe COVID-19 disease**

**
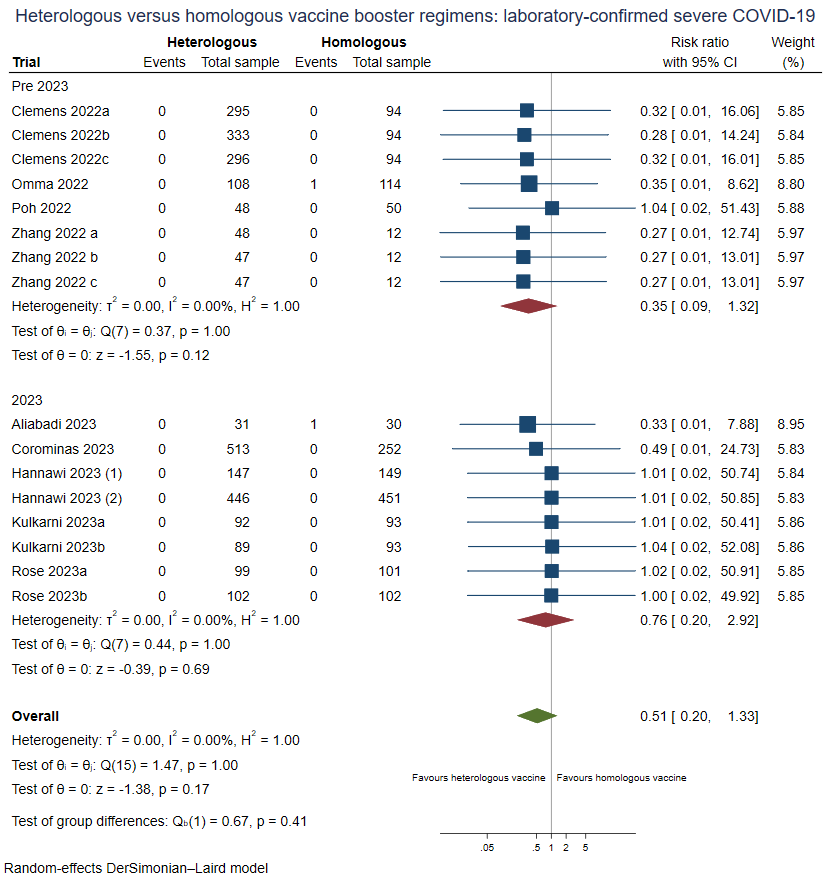
**

*Figure S33 Subanalysis for laboratory-confirmed severe COVID-19 by pre 2023 and in 2023 to capture the predominance of Omicron subvariant XBB.*

**Laboratory-confirmed symptomatic COVID-19**

**
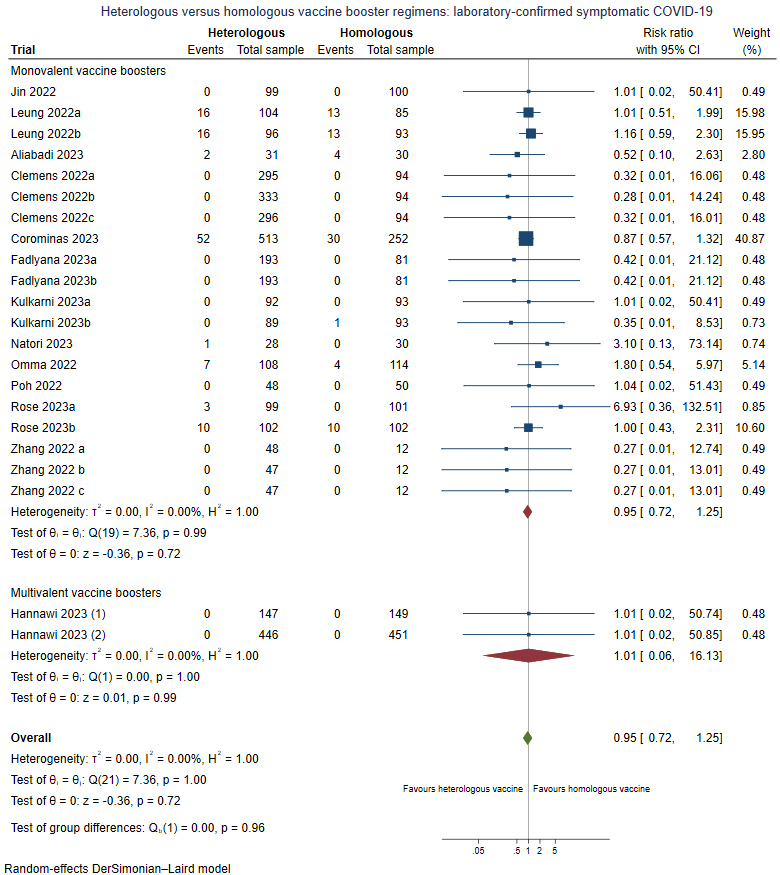
**

*Figure S34 Subanalysis for laboratory-confirmed symptomatic COVID-19 according to vaccine booster valency.*

**Laboratory-confirmed severe COVID-19**

**
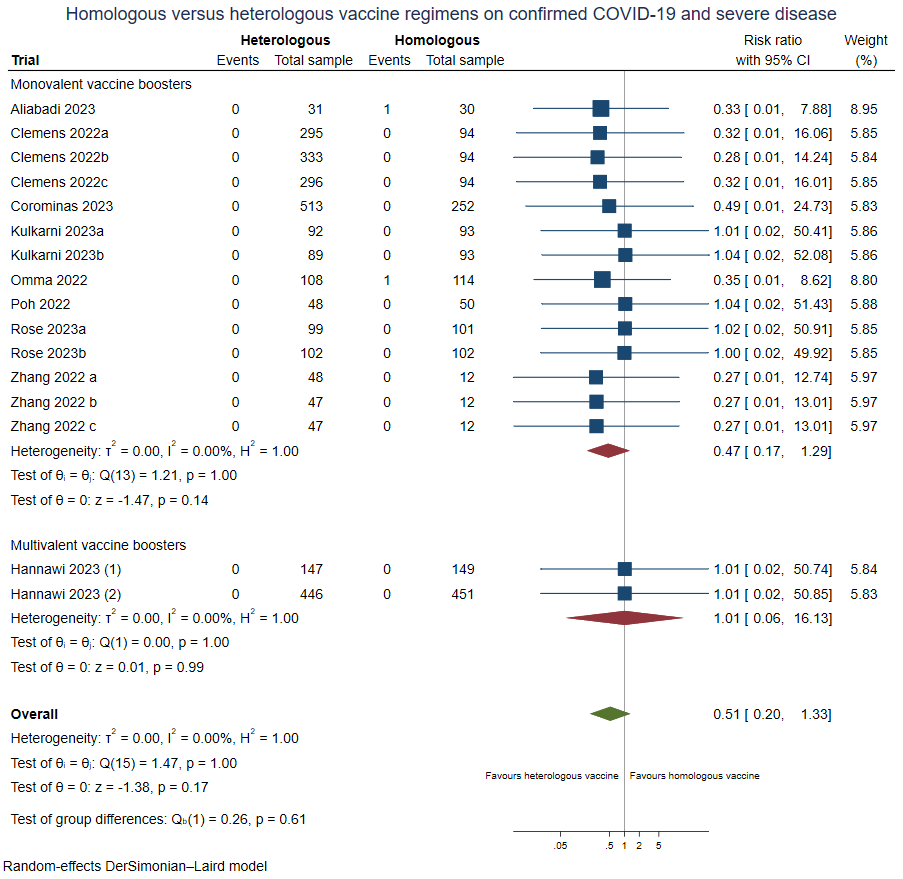
**

*Figure S35 Subanalysis for laboratory-confirmed severe COVID-19 according to vaccine booster valency.*

**Serious adverse events**

**
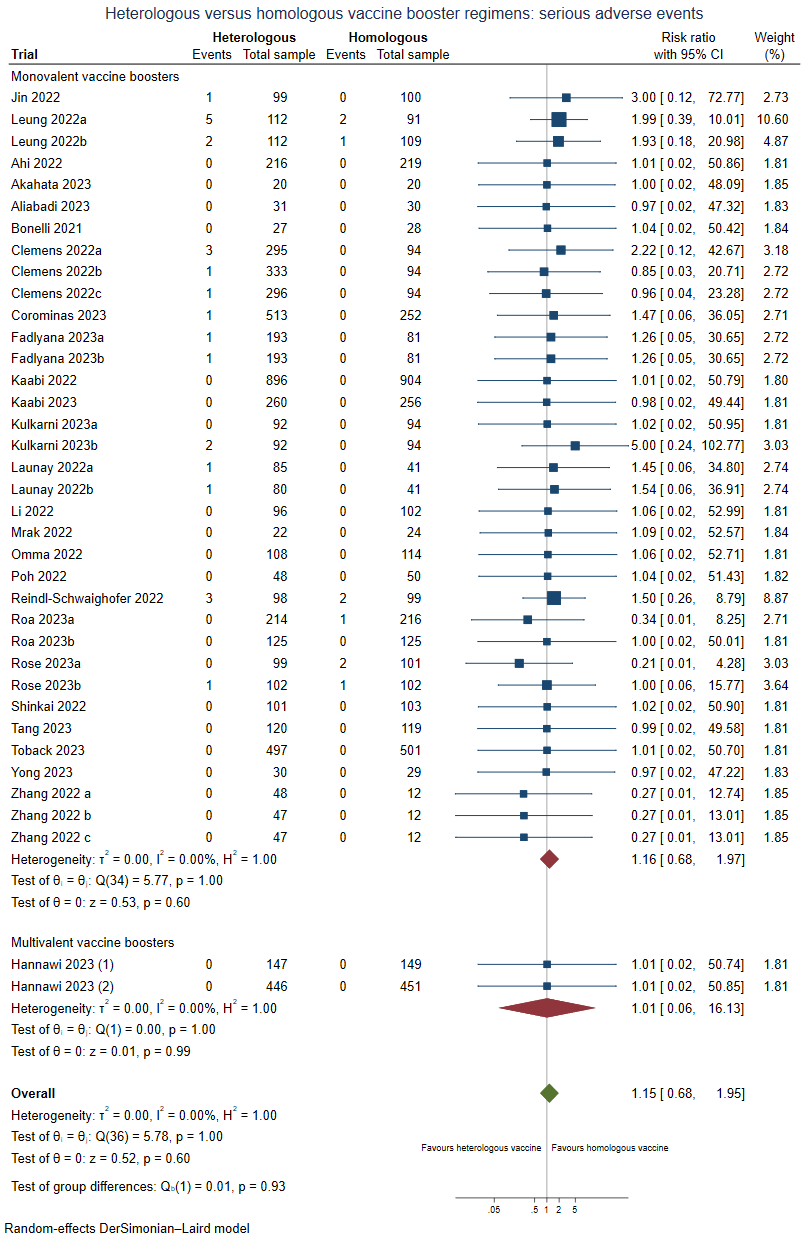
***Figure S36 Subanalysis for SAE according to vaccine booster valency*

## Publication bias

**
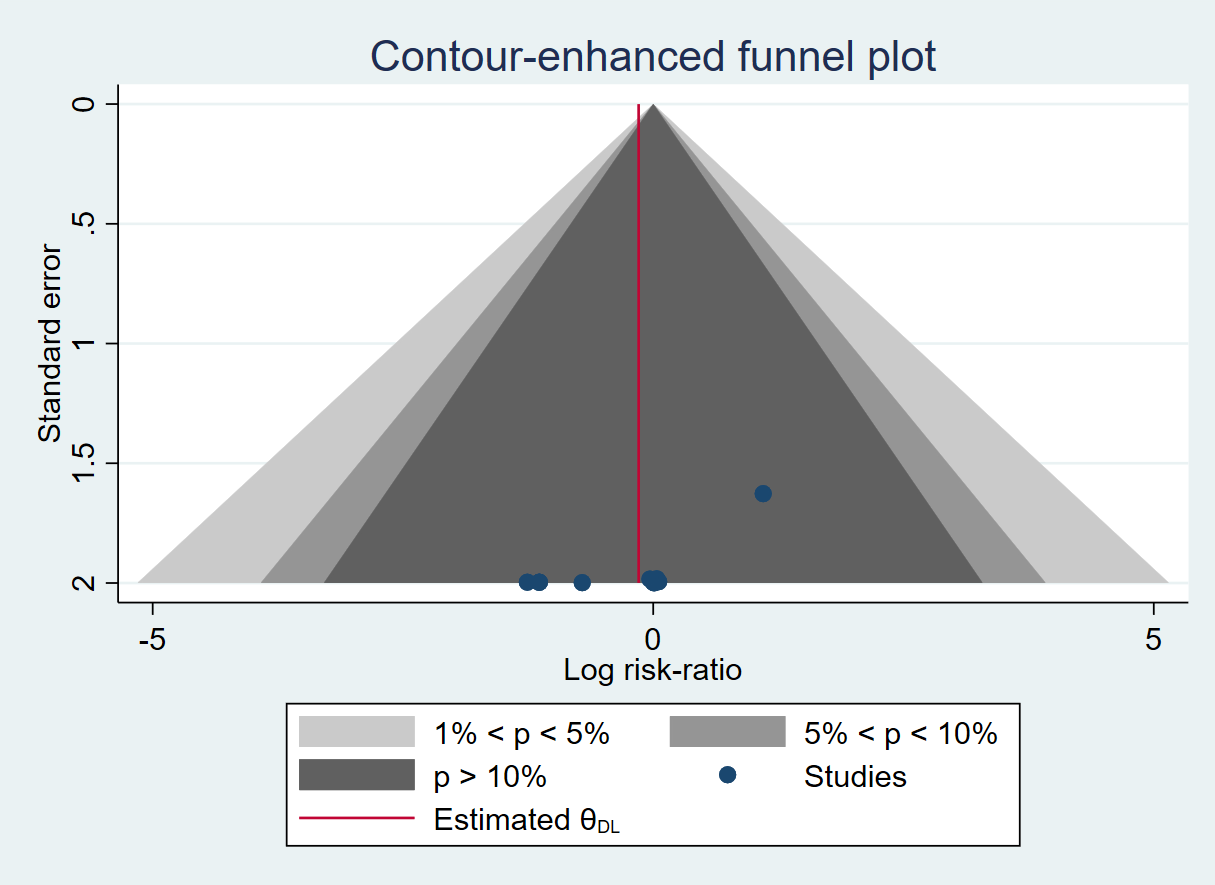
**

*Figure S37 All-cause mortality*

**
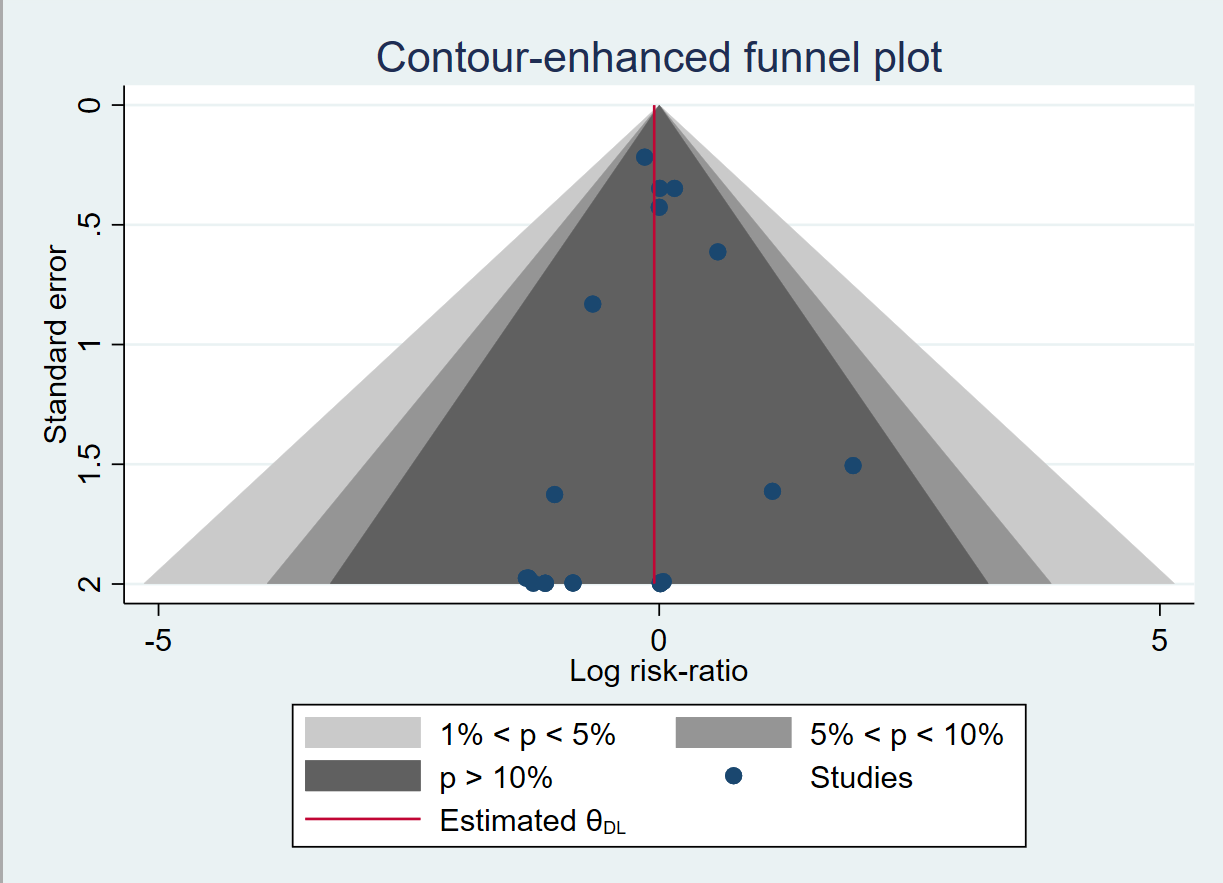
**

*Figure S38 Funnel plot for laboratory-tested symptomatic COVID-19*

**
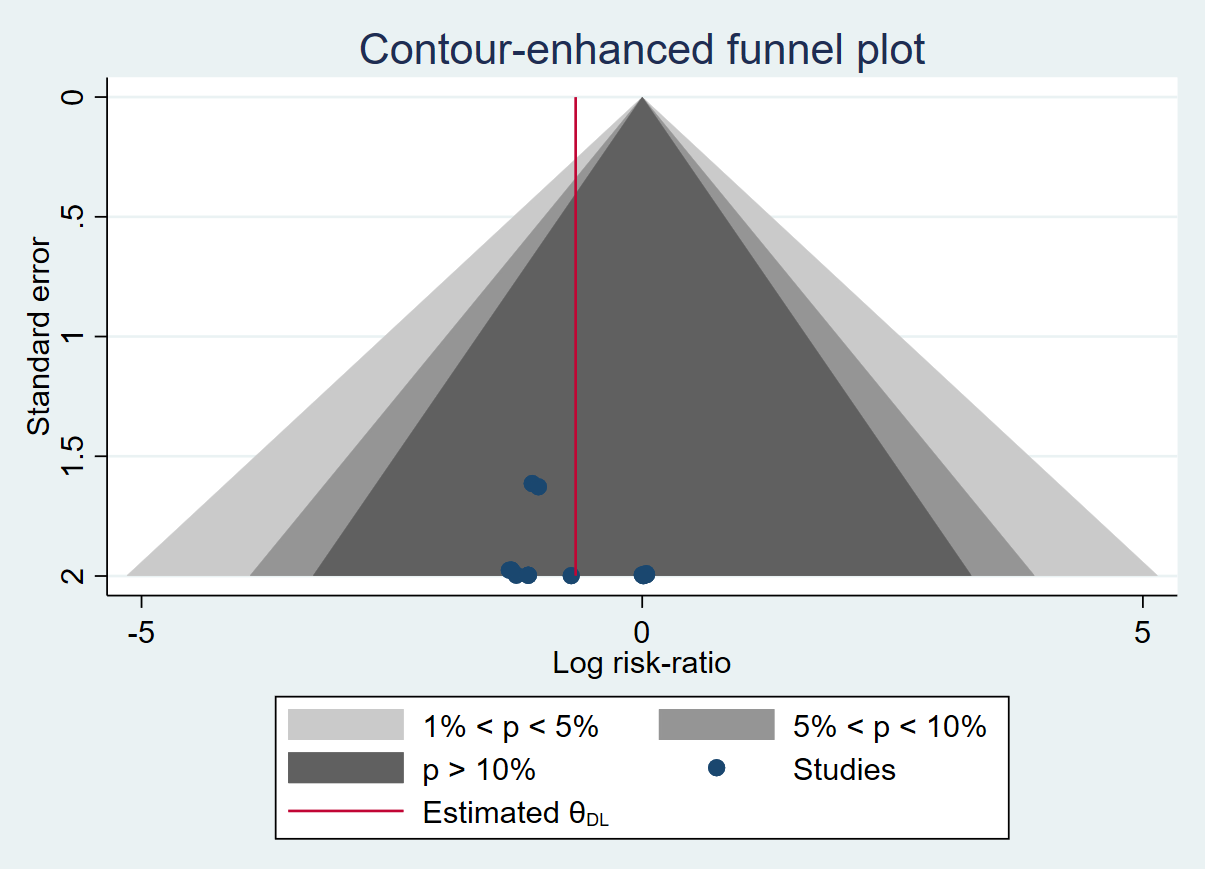
**

*Figure S39: Funnel plot for laboratory-confirmed severe COVID-19*

**
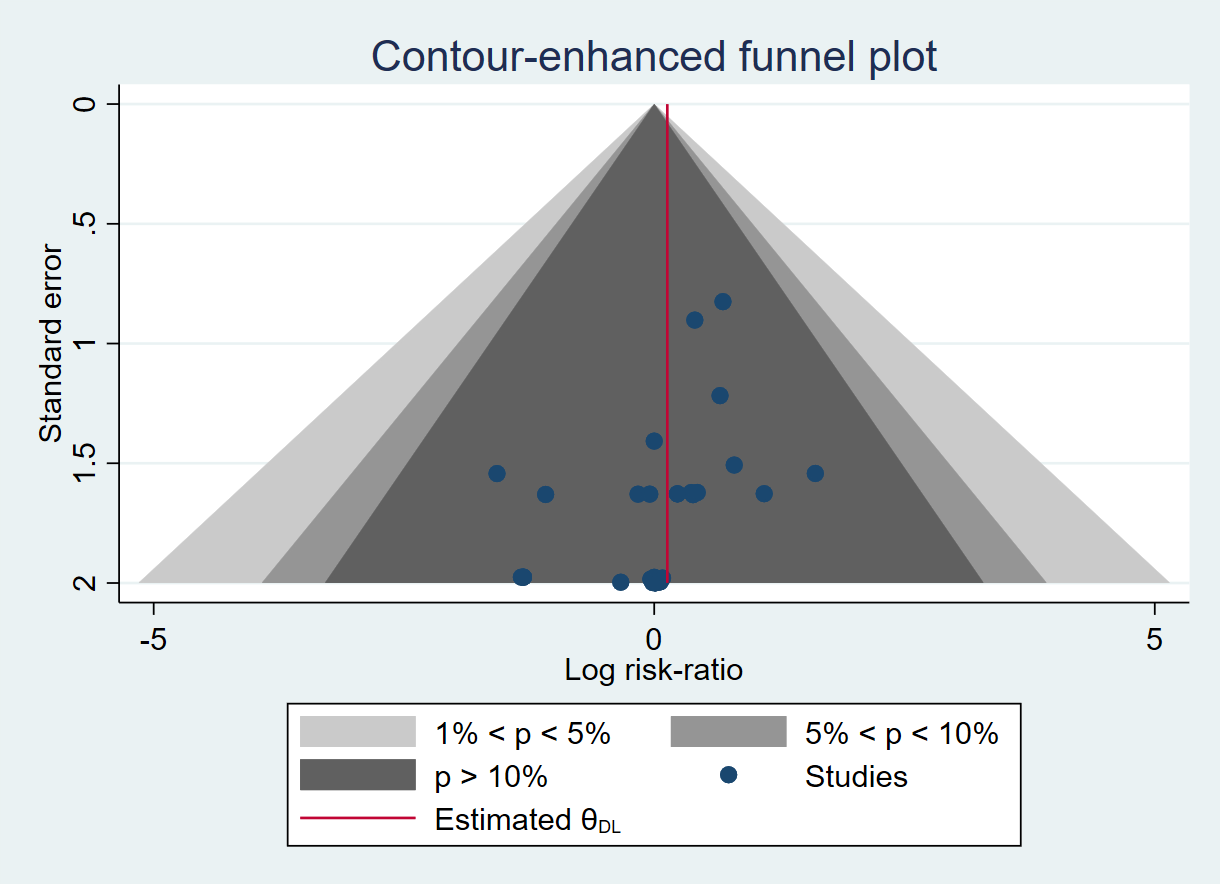
**

*Figure S40: Funnel plot for serious adverse events*

**
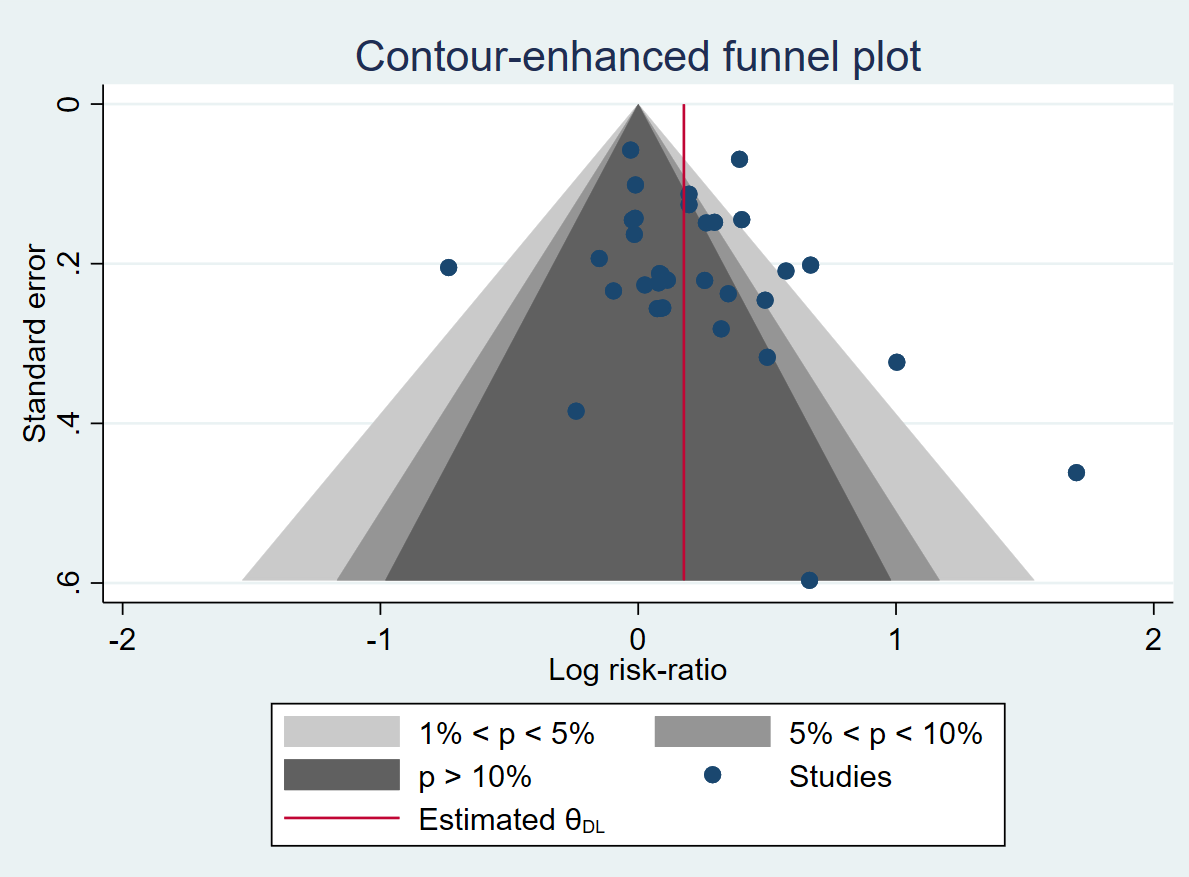
**

*Figure S41: Funnel plot for adverse events considered non serious*

**Regression-based Egger test for small-study effects**

**All-cause mortality**

Egger Test Results: Estimated beta1 = -3.79; Standard Error of beta1=4.64; z-statistics= -0.82; P value: 0.4

**Laboratory-confirmed symptomatic COVID-19**

Egger Test Results: Estimated beta1 = -0.19; Standard Error of beta1=0.30; z-statistics= --0.64; P value: 0.52

**Laboratory-confirmed severe COVID-19**

Egger Test Results: Estimated beta1 = 1.44; Standard Error of beta1=3.4; z-statistics= 0.42; P value: 0.68

**Serious adverse events**

Egger Test Results: Estimated beta1 = -0.68; Standard Error of beta1=0.64; z-statistics= -1.05; P value: 0.29

**Adverse events considered non-serious**

Egger Test Results: Estimated beta1 =1.42; Standard Error of beta1=0.63; z-statistics=2.26; P value: 0.02

**PRISMA flow chart**


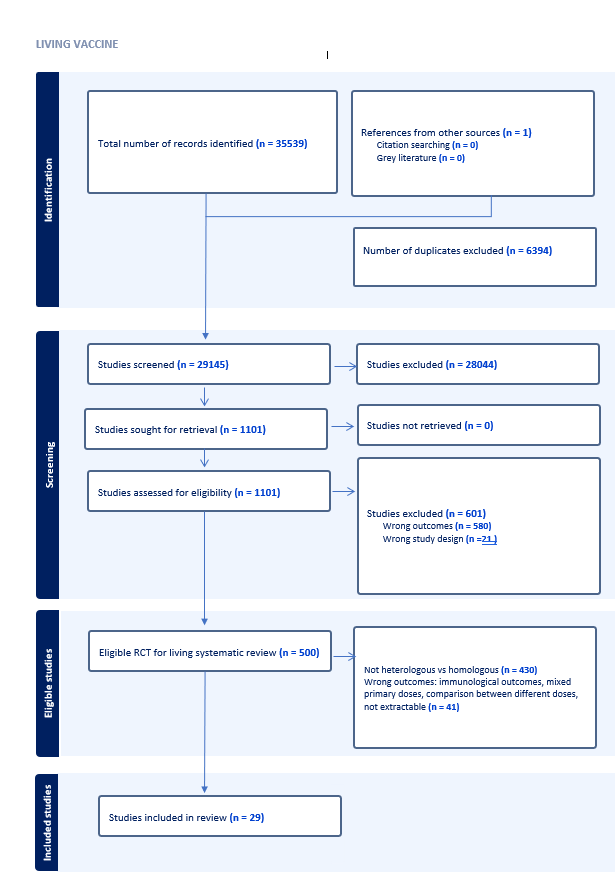


*Figure: S42 PRISMA flow chart*


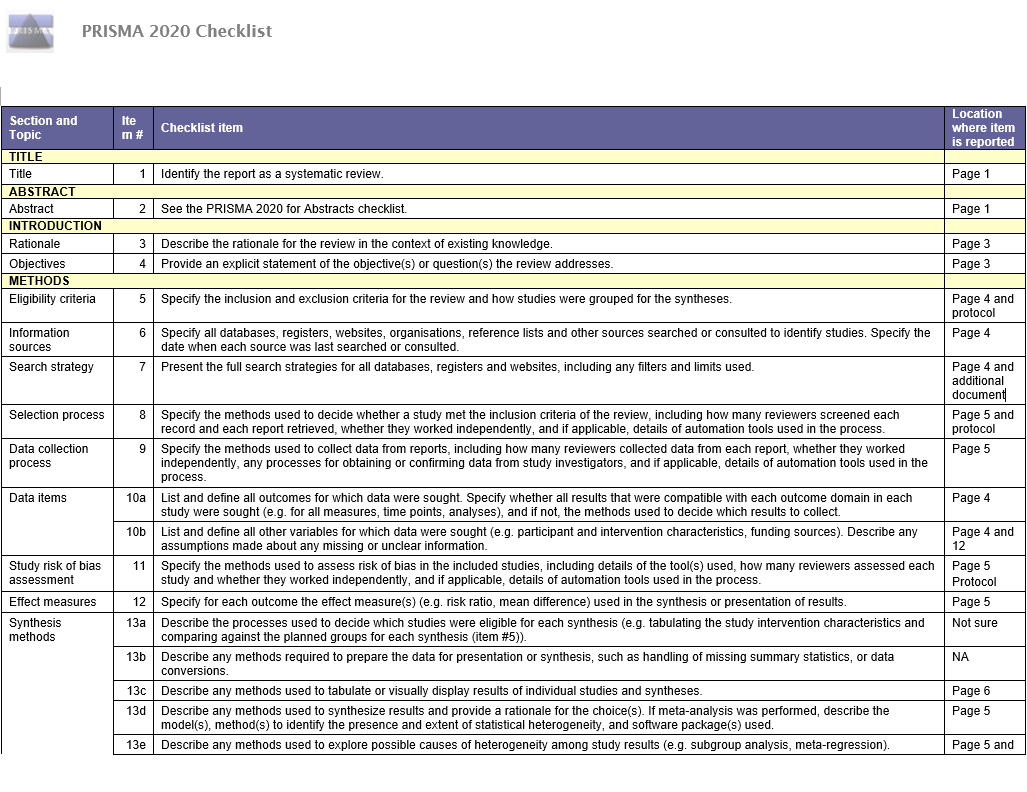


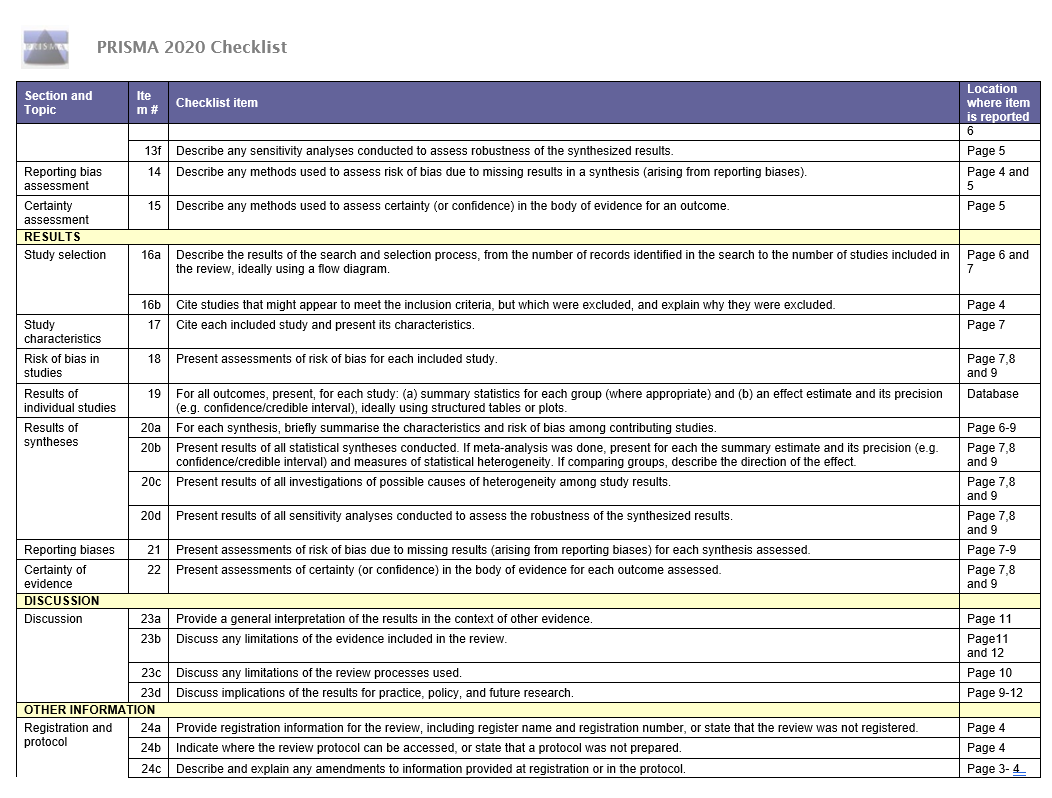


*Figure: S43 PRISMA checklist*
